# ACSS3 represses prostate cancer progression through downregulating lipid droplet-associated protein PLIN3

**DOI:** 10.7150/thno.49384

**Published:** 2021-01-01

**Authors:** Lijie Zhou, Zhengshuai Song, Junyi Hu, Lilong Liu, Yaxin Hou, Xiaoping Zhang, Xiong Yang, Ke Chen

**Affiliations:** 1 Department of Urology, Union Hospital, Tongji Medical College, Huazhong University of Science and Technology, Wuhan, Hubei, China;; 2 Shenzhen Huazhong University of Science and Technology Research Institute, Shenzhen, China;; 3 Department of Urology, The Central Hospital of Wuhan, Tongji Medical College, Huazhong University of Science and Technology, Wuhan, China

**Keywords:** prostate cancer, ACSS3, PLIN3, lipid droplet

## Abstract

Current endocrine therapy for prostate cancer (PCa) mainly inhibits androgen/androgen receptor (AR) signaling. However, due to increased intratumoural androgen synthesis and AR variation, PCa progresses to castration-resistant prostate cancer (CRPC), which ultimately becomes resistant to endocrine therapy. A search for new therapeutic perspectives is urgently needed.

**Methods:** By screening lipid metabolism-related gene sets and bioinformatics analysis in prostate cancer database, we identified the key lipid metabolism-related genes in PCa. Bisulfite genomic Sequence Polymerase Chain Reaction (PCR) (BSP) and Methylation-Specific Polymerase Chain Reaction (PCR) (MSP) were preformed to detect the promoter methylation of ACSS3. Gene expression was analyzed by qRT-PCR, Western blotting, IHC and co-IP. The function of ACSS3 in PCa was measured by CCK-8, Transwell assays. LC/MS, Oil Red O assays and TG and cholesterol measurement assays were to detect the levels of TG and cholesterol in cells. Resistance to Enzalutamide in C4-2 ENZR cells was examined in a xenograft tumorigenesis model in vivo.

**Results:** We found that acyl-CoA synthetase short chain family member 3 (ACSS3) was downregulated and predicted a poor prognosis in PCa. Loss of ACSS3 expression was due to gene promoter methylation. Restoration of ACSS3 expression in PCa cells significantly reduced LD deposits, thus promoting apoptosis by increasing endoplasmic reticulum (ER) stress, and decreasing de novo intratumoral androgen synthesis, inhibiting CRPC progression and reversing Enzalutamide resistance. Mechanistic investigations demonstrated that ACSS3 reduced LD deposits by regulating the stability of the LD coat protein perilipin 3 (PLIN3).

**Conclusions:** Our study demonstrated that ACSS3 represses prostate cancer progression through downregulating lipid droplet-associated protein PLIN3.

## Introduction

Prostate cancer (PCa) is an androgen-dependent tumor, and androgen can stimulate PCa cell growth and disease progression. Traditional endocrine therapies include androgen deprivation therapy (ADT) that blocks testicular androgens and antiandrogen therapy that block adrenal androgens 1-2. ADT and antiandrogen therapy are the main methods of clinical interventions in advanced PCa. Although a majority of patients initially respond to ADT and antiandrogen therapy well, some of them will eventually develop castration-resistant PCa (CRPC) because of reactivation and abnormal activation of androgen receptor (AR) 3. In the CRPC stage, Abiraterone (inhibitor of the enzyme CYP17A1 required for androgen synthesis) and Enzalutamide (more effective direct AR antagonist) in CRPC established that further AR suppression can extend patient survival 4-6. However, due to continuous AR variants, CRPC ultimately does not respond to Enzalutamide or Abiraterone 7. AR splice variants encode a truncated AR protein that lacks the ligand-binding domain and is constitutively active in the absence of androgens. AR-V7 is the most common AR splice variant. The androgen-receptor isoform encoded by splice variant 7 lacks the ligand-binding domain, which is the target of enzalutamide and abiraterone, but remains constitutively active as a transcription factor 57-58. A search for new therapeutic perspectives is urgently needed.

Abnormal lipid accumulation has been found in many tumor cells, such as clear cell renal carcinoma, breast cancer and PCa 8-10. Some studies have shown that lipid accumulation in PCa cells is closely related to the development of CRPC and Enzalutamide or Abiraterone resistance 3, 11. AR signaling-mediated lipid accumulation in PCa cells increases intratumoral androgen synthesis, which promotes AR reactivation and abnormal activation, leading to CRPC progression 11-13. In the CRPC stage, AR-V7 increases lipid accumulation via acyl-CoA synthetase long chain family member 3 (ACSL3) and membrane bound O-acyltransferase domain containing 2 (MBOAT2), thereby mediating resistance to Enzalutamide 14. Effective ways to reduce lipid accumulation in PCa remain unclear.

Abnormal accumulation of lipids in cells usually occurs in the form of lipid droplets (LDs). LDs consist of a neutral lipid core containing triglycerides and cholesterol esters surrounded by a phospholipid monolayer and associated LD surface proteins. In the present study, we found that ACSS3/PLIN3 signaling inhibits PCa progression through increasing ER stress and reverses Enzalutamide resistance by reducing intratumoral LD deposits. Mechanistic investigations demonstrated that ACSS3 reduced LD deposits by regulating the stability of the LD coat protein PLIN3. Together, these results establish ACSS3 inhibits prostate cancer progression and new endocrine therapy resistance by reducing intratumoral lipid accumulation.

## Results

### ACSS3 was downregulated and predicted poor prognosis in prostate cancer

**Figure 1:**
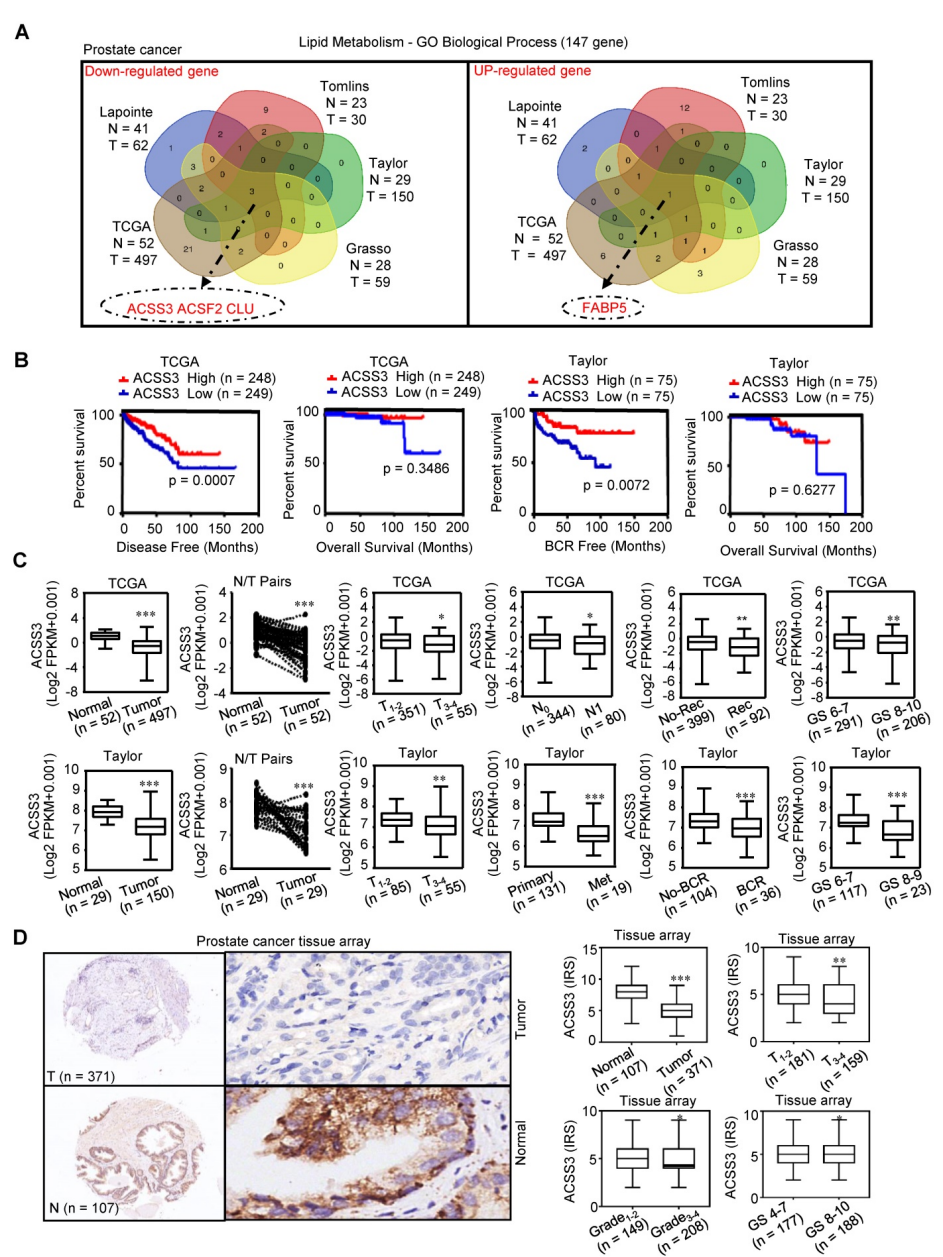
** ACSS3 was downregulated and predicted poor prognosis in PCa.** (A) By screening lipid metabolism‐related gene sets in five independent PCa databases, four genes were selected; three of these genes (ACSS3, ACSF2 and CLU) were found to be downregulated and one gene (FABP5) was found to be upregulated (*│Fold change│>1.5,* p < *0.05*). (B) Kaplan-Meier analysis in the TCGA and Taylor databases was conducted to determine whether the OS, DFS and BRFS of patients were associated with ACSS3 gene expression in tumors. (C) According to in-depth analysis of the TCGA and Taylor databases, low ACSS3 expression was positively correlated with tumor stage, metastasis, recurrence and Gleason score. *Student's t* test; *, p < *0.05*; **, p < *0.01*; ***, p < *0.001.* (D) ACSS3 protein expression was analyzed using PCa tissue arrays from 107 normal tissues and 371 tumor tissues. ACSS3 protein levels were significantly lower in PCa tissues than in normal tissues. Low ACSS3 expression was positively correlated with tumor stage, grade and Gleason score.* Student's t* test; *, p < *0.05*; **, p < *0.01*; ***, p < *0.001.*

Lipid metabolism is significantly dysregulated in PCa 47. By screening lipid metabolism‐related gene sets in five independent PCa databases (TCGA Prostate, Taylor Prostate, Lapointe Prostate, Tomlins Prostate, Grasso Prostate), four genes were selected; three of these genes (ACSS3, ACSF2 and CLU) were found to be downregulated and one gene (FABP5) was found to be upregulated in PCa compared to normal prostate tissues (/*FC/ > 1.5,* p < *0.05*) (Figure 1A). A Kaplan-Meier analysis of data in the TCGA and Taylor databases was conducted to determine whether the overall survival (OS), disease-free survival (DFS) and biochemical relapse-free survival (BRFS) of patients were associated with the expression of the four selected genes in tumors. Gene expression was used to assign patients to a high (above the 50^th^ percentile) or low (below the 50^th^ percentile) expression group. As shown in Figure 1B and Figure S1A, the patients with tumors that only expressed high levels of ACSS3 had longer DFS (p = 0.0007) and BRFS (p = 0.0072) than patients with tumors expressing low levels of ACSS3. Then, receiver operating characteristic (ROC) curves were used to analyze the diagnostic value of ACSS3 in PCa in the TCGA and Taylor databases. As shown in Figure S1B, ACSS3 had a high area under the curve (AUC) value in PCa patients, indicating that ACSS3 could effectively distinguish PCa tissues from normal tissues. Next, according to an in-depth analysis of the TCGA and Taylor databases, we found that low ACSS3 expression was positively correlated with tumor stage, metastasis, recurrence and Gleason score (Figure 1C) and served as an independent prognostic factor (Table S2). The results of Table S2 indicated that cancer patients with lower ACSS3 expression had significantly shorter disease-free time, in both multivariate analysis (HR 0.575, 95% CI 0.36-0.91, P = 0.018) and univariate analysis (HR 0.563, 95% CI 0.36-0.89, P = 0.013), as shown in Table S1. Besides, univariate analysis showed that shorter disease-free time was significantly correlated with a more advanced T and N stage (HR 1.65, 95% CI 0.99-2.73, p = 0.05), higher level of PSA (HR 2.90, 95% CI 1.33-6.33, p = 0.007), while there was no significant correlation between disease-free time and M stage (HR 0.049, 95% CI 0.00-7.30, p = 0.72). Meanwhile, recurrence and higher Gleason score was significantly correlated with a shorter disease-free time, according to both multivariate analysis and univariate analysis.

To further confirm the results, we examined ACSS3 protein levels by immunohistochemical (IHC) staining in PCa specimens. Similar to our previous result, we observed that ACSS3 protein levels were significantly lower in the PCa tissues (n = 371) than in the normal tissues (n = 107) (Figure 1D and Table S3). Low ACSS3 expression was positively correlated with tumor stage and Gleason score (Figure 1D and Table S3). Collectively, these results indicated that ACSS3 was downregulated and predicted poor prognosis in prostate cancer.

### Loss of ACSS3 expression was due to gene promoter methylation

To explore the mechanism of ACSS3 downregulation in PCa, we analyzed the genomic DNA sequence within the 2-kilobase promoter regions of the ACSS3 gene and found that the ACSS3 gene contains CpG-rich regions (CpG islands) within the promoter regions (Figure S2A). The publicly available Cancer Cell Line Encyclopedia (CCLE) database was used for bioinformatic analyses to evaluate the methylation status of the ACSS3 promoter in different PCa cell lines. Methylation was enriched in the ACSS3 promoter (Figure S2B). When comparing the methylation status of the ACSS3 promoter in PCa cells and in normal prostate cells by bisulfite sequencing PCR (BSP) analysis, we found that significant hypermethylation of the ACSS3 promoter was found in PCa cells compared with normal prostate cells (Figure S2C). Based on the results of BSP analysis, we also confirmed the methylation sites within the ACSS3 promoter. Next, we conducted a methylation-specific PCR (MSP) assay to verify the methylation sites of the ACSS3 promoter in different PCa cell lines (Figure S2D). The results of western bolt verified that the protein levels of the ACSS3 in PCa lines was lower than the prostate epithelial cell line (RWPE-1) (Figure S2E). To further determine whether DNA methylation is responsible for ACSS3 downregulation, we treated 22RV1, PC3 and C4-2 cells with or without 5-aza-dC, a demethylation reagent. Demethylation via 5-aza-dC treatment reduced the methylation levels of the ACSS3 promoter and increased ACSS3 mRNA and protein expression levels in PCa cells in a dose-dependent manner (Figure S2F), indicating that hypermethylation plays a crucial role in the silencing of ACSS3 expression. Finally, we analyzed the ACSS3 promoter methylation status in PCa samples. The BSP results showed that the methylation levels of ACSS3 promoter regions in tumors were higher than those in paired normal tissues (Figure S2G). Collectively, these results indicated that DNA hypermethylation may be the main reason for ACSS3 downregulation in PCa.

### ACSS3 inhibited abnormal lipid accumulation and tumor cell growth

**Figure 2:**
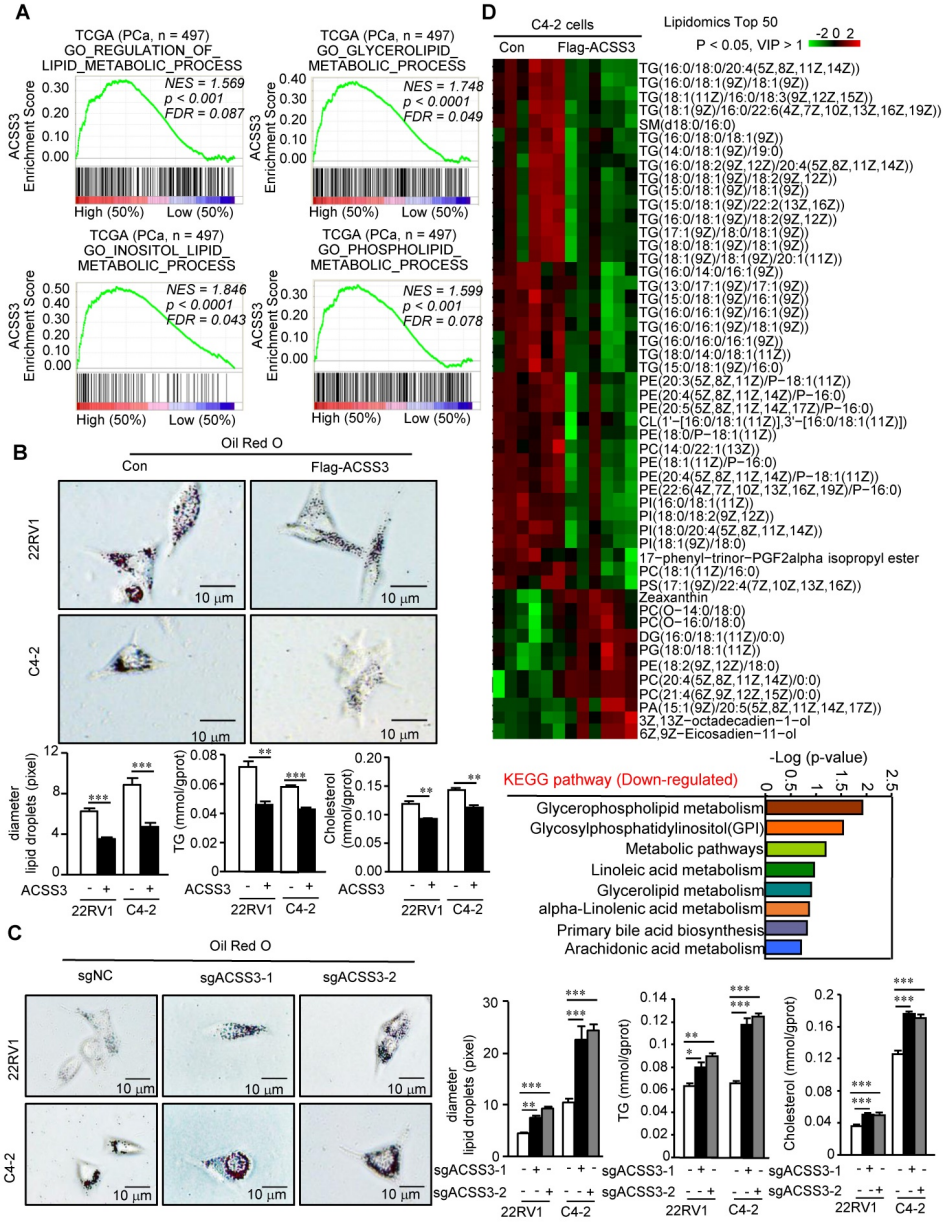
** ACSS3 significantly reduced the abnormal lipid accumulation in PCa cells.** (A) GSEA of ACSS3 mRNA and PCa signaling pathways. *FDR < 25%* and p < *0.05* were considered significant. (B-C) ACSS3-overexpressing or ACSS3-knockout PCa cell lines were generated by transfecting overexpressing lentivirus and sgRNA (CRISPR-Cas9). Oil Red O staining was conducted as a visual indicator of intracellular lipids in PCa. The relative diameters of LDs and TG and cholesterol contents were measured as quantitative indicators of lipid accumulation in PCa cells. The results represent the mean ± SEM from 3 independent experiments. (D) An LC/MS lipidomic assay was conducted to detect intracellular lipids in C4-2 cells with or without stable overexpression of ACSS3 (n = 6). Only statistically significant changes (Student's t test, p < *0.05, VIP > 1*) are presented, indicated by the red and green colors in the heat map. Downregulated KEGG pathways for lipid metabolism based on the results from the LC/MS lipidomic assay.

Next, we performed gene set enrichment analysis (GSEA) to analyze the gene sets altered by ACSS3 expression in the PCa TCGA dataset. Our results showed that pathways related to the suppression of PCa and metastasis and pathways related to lipid metabolic processes were highly associated with high ACSS3 expression (above the 50^th^ percentile) (Figure 2A and Figure S3A), suggesting that ACSS3, as a tumor suppressor, is involved in the regulation of tumor growth, metastasis and lipid metabolism. To test this hypothesis, we successfully generated C4-2 and 22RV1 cell lines with overexpression or knockout of ACSS3 by using lentivirus or sgRNA (CRISPR-Cas9), respectively (Figure S3B**)**. CCK-8, migration and invasion assays were conducted. The results showed that cells stably overexpressing ACSS3 had significantly reduced proliferation, migration and invasion, while ACSS3-knockout cell lines tended to display increased proliferation, migration and invasion (Figure S3C-E). We used PC3 cells to detect the effect of ACSS3 in AR negative prostate cancer, and the results showed that ACSS3 could also inhibit the proliferation of PC3 cells (Figure S3G). Furthermore, to clarify the regulation of lipid metabolism mediated by ACSS3, an oil red staining assay was conducted as a visual indicator of intracellular lipids in PCa. The results showed that LD deposits was obviously decreased in PCa cell lines overexpressing ACSS3, while a significant increase in LD deposits was observed in the ACSS3-knockout cell lines (Figure 2B-C). Subsequently, triglyceride (TG) and cholesterol contents were measured as quantitative indicators of lipid accumulation in PCa. As shown in Figure 2B-C, the cells with stable overexpression of ACSS3 exhibited relatively lower TG and cholesterol contents, while the cells with stable knockout of ACSS3 exhibited relatively higher TG and cholesterol contents. Finally, we conducted a liquid chromatography/mass spectrometry (LC/MS) lipidomic assay by comparing C4-2 cells with or without ACSS3 overexpression. As shown in Figure 2D and Figure S3F, the important components of LDs, namely, TG, phosphatidylethanolamine (PE), phosphatidylinositol (PI) and phosphatidylcholine (PC), were significantly downregulated in stable ACSS3-overexpressing C4-2 cells compared to control cells.

### ACSS3 promoted ER stress-mediated cell apoptosis

**Figure 3:**
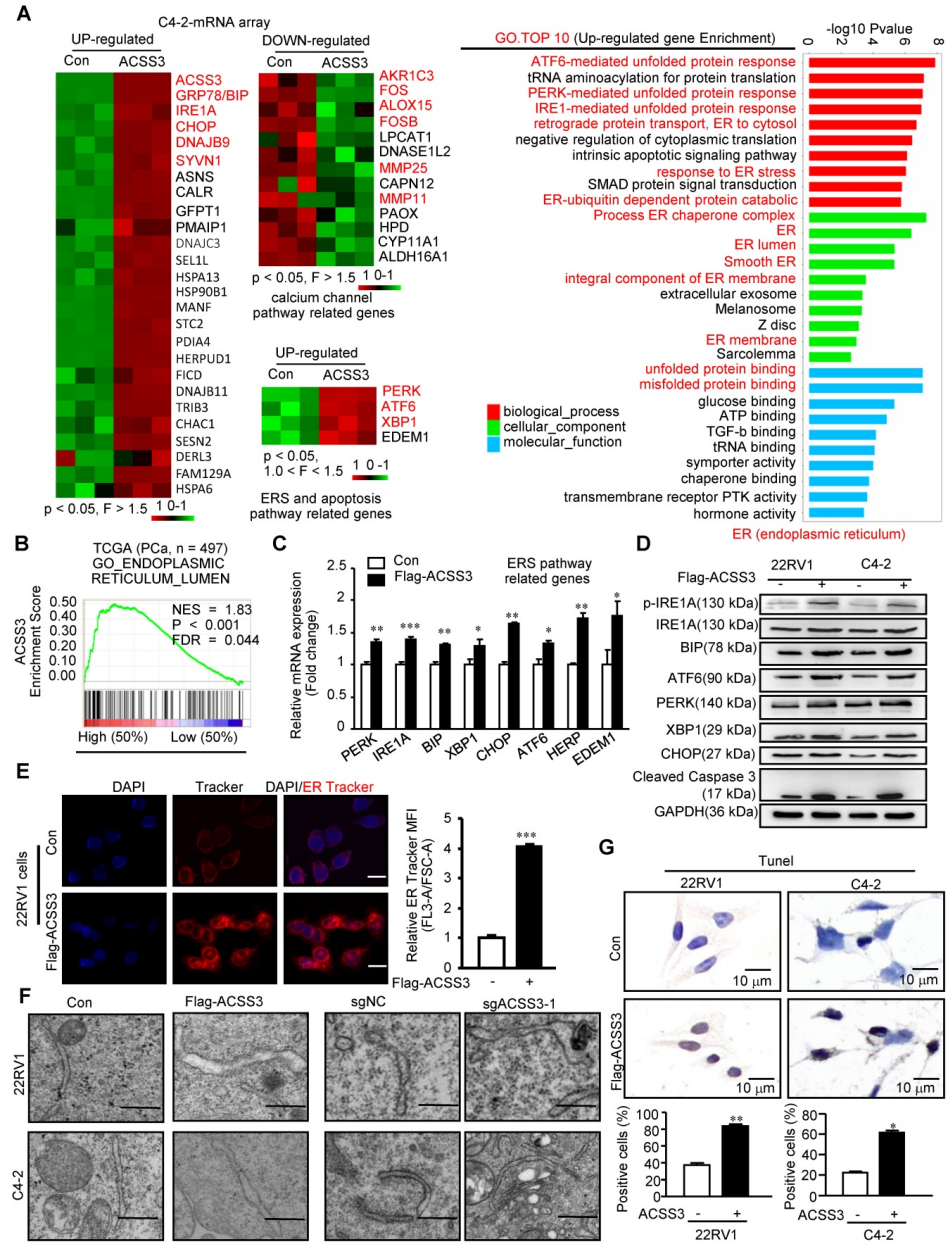
** ACSS3 promoted ER stress-mediated cell apoptosis.** (A) The microarray assays were conducted in C4-2 cells with stable overexpression of ACSS3 (n = 3). GO analysis showed that ACSS3 overexpression significantly upregulated ER stress-, unfolded protein- and misfolded protein-related signaling pathways. (B) GSEA of ACSS3 mRNA and PCa signaling pathways. *FDR < 25%* and p < *0.05* were considered significant. (C-D) qRT-PCR and Western blot analysis were performed to validate the microarray data. (E) ER Track imaging indicated ER stress expansion in ACSS3-overexpressing 22RV1 cells. The bar is 10 μm. (F) Ultrastructural analysis by transmission electron microscopy (TEM) confirmed the presence of dilated and irregularly shaped rough ER in ACSS3-overexpressing C4-2 and 22RV1 cells or ACSS3-knockout cells. The bar is 0.5 μm. (G) TUNEL assays were performed to detect cell apoptosis. The results represent the mean ± SEM from 3 independent experiments. *Student's t* test; *, p < *0.05*; **, p < *0.01*; ***, p < *0.001.*

To determine the regulatory mechanisms of ACSS3 in PCa, we first performed the microarray assay in C4-2 cells stably overexpressing ACSS3. Previous studies have demonstrated that LD deposits suppress the cytotoxic endoplasmic reticulum (ER) stress response and promote ER homeostasis and cell viability in tumor cells 15. As expected, sequencing results revealed that overexpression of ACSS3 in C4-2 cells resulted in a significant increase in the ER stress markers GRP78 (BIP), IREA, CHOP, DNAJB9, SYVN1, PERK, ATF6 and XBP1 and a significant decrease in the calcium channel pathway markers AKR1C3, FOS, ALOX15, FOSB, MMP25 and MMP11 (Figure 3A).

Subsequently, GO analysis showed that ACSS3 overexpression significantly upregulated the ER stress-, unfolded protein- and misfolded protein-related signaling pathways and significantly downregulated the calcium channel-related signaling pathway (Figure 3A and Figure S4A). ER stress is characterized by misfolded and unfolded protein accumulation and calcium balance disturbance in the ER lumen. GSEA indicated that ACSS3 was also highly associated with the ER lumen signaling pathway in the TCGA database (Figure 3B). To validate the microarray data, we performed qRT-PCR and Western blot analyses. Overexpression of ACSS3 in 22RV1 and C4-2 cells was associated with increased ER stress markers (Figure 3C-D and Figure S4B). Conversely, knockout of ACSS3 in 22RV1 and C4-2 cells resulted in decreased expression of ER stress markers (Figure S4C-D). Furthermore, ER Track imaging analysis indicated ER expansion in ACSS3-overexpressing cells (Figure 3E), and ultrastructural analysis by transmission electron microscopy (TEM) confirmed the presence of dilated and irregularly shaped rough ER (Figure 3F), both of which are consistent with ER stress. Since ER stress often activates the apoptotic signaling pathway 48, we performed TUNEL assays to detect cell apoptosis. As shown in Figure 3G, Overexpression of ACSS3 in 22RV1 and C4-2 cells was associated with an increased percentage of apoptotic cells. Conversely, ACSS3-knockout cell lines tended to display decreased apoptosis (Figure S4E). Taken together, these results indicated that ACSS3 promoted ER stress-mediated cell apoptosis.

### ACSS3 regulated the protein stability of PLIN3

**Figure 4:**
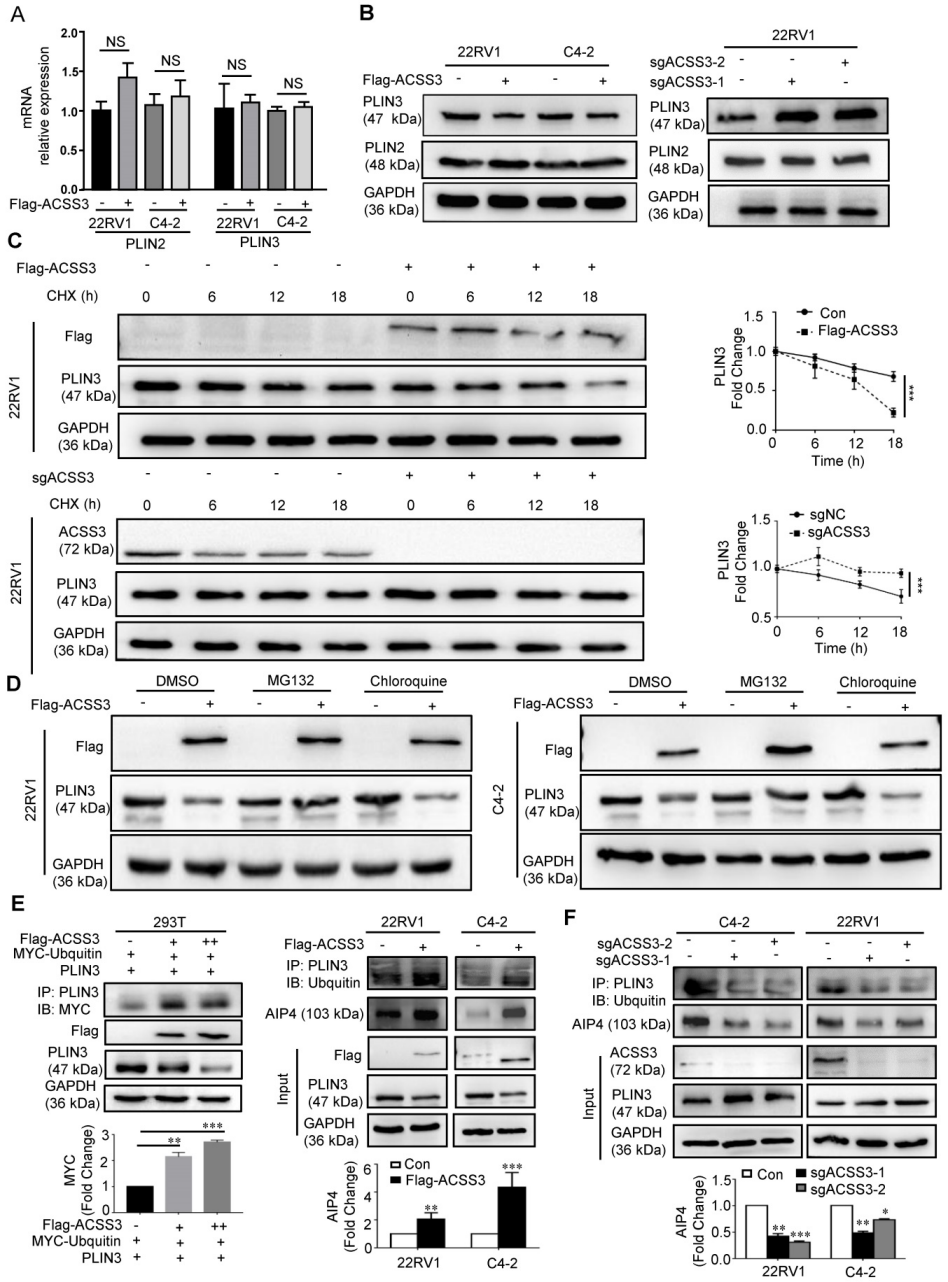
** ACSS3 regulated PLIN3 protein stability by affecting PLIN3 ubiquitination levels.** (A) PLIN2 and PLIN3 mRNA levels were determined by qRT-PCR in C4-2 and 22RV1 cells with stable ACSS3 overexpression. The results represent the mean ± SEM from 3 independent experiments. Student's t test; ns, no significance. (B) PLIN2 and PLIN3 protein levels were determined by Western blot analysis in C4-2 and 22RV1 cells with stable ACSS3 overexpression, and 22RV1 cells with stable knockout of ACSS3. One representative experiment of 3 independent experiments is shown. (C) 22RV1 cells stably overexpressing ACSS3 were treated with cycloheximide (CHX, 10 μmol/L) to inhibit protein synthesis, and PLIN3 protein turnover was analyzed over time. Similarly, 22RV1 cells with stable knockout of ACSS3 were also treated with CHX. And statistic analysis. One representative experiment of 3 independent experiments is shown. (D) C4-2 and 22RV1 cells stably overexpressing ACSS3 were treated with proteasome inhibitor (MG132, 20 μM) or lysosome inhibitor (chloroquine, 50 μM). Western bolt was performed to detect the level of PLIN3. One representative experiment of 3 independent experiments is shown. (E) PLIN3‐related immunoprecipitation was used to assess the levels of ubiquitinated PLIN3 and AIP4 in C4-2 and 22RV1 cells stably overexpressing ACSS3, and 293T cells stably overexpressing ACSS3, PLIN3 and Myc-Ubquin. (F) PLIN3‐related immunoprecipitation was used to assess the levels of ubiquitinated PLIN3 and AIP4 in C4-2 and 22RV1 cells with stable knockout of ACSS3. One representative experiment of 3 independent experiments is shown.

Perilipin family proteins (PLINs) are the predominant LD-associated proteins and include PLIN1, PLIN2, PLIN3, PLIN4 and PLIN5. In mammals, PLIN1 and PLIN4 is primarily expressed in adipose tissues and is a major regulator of lipolysis in adipocytes 16, 64. PLIN5 is expressed in oxidative tissues like skeletal muscle 65. PLIN2 and PLIN3, as regulators of LD biogenesis and degradation, help coat LDs in most other cell types 17-18. Next, we used qRT-PCR and Western blot to examine the mRNA and protein levels of PLIN2 and PLIN3 in 22RV1 and C4-2 cells with overexpression or knockout of ACSS3. The qRT-PCR results showed no statistically significant changes in the mRNA levels of PLIN2 and PLIN3 (Figure 4A). However, overexpression of ACSS3 in C4-2 and 22RV1 cells resulted in decreased PLIN3 protein levels, and knockout of ACSS3 in 22RV1 cells resulted in increased PLIN3 protein levels (Figure 4B and Figure S5D) compared to those in control cells. The inconsistency between the protein and mRNA levels of PLIN3 led us to suggest that ACSS3 may control PLIN3 levels by regulating the stability of the PLIN3 protein. Accordingly, 22RV1 cells stably overexpressing ACSS3 or with stable knockout of ACSS3 were treated with cycloheximide (CHX) to inhibit protein synthesis, and PLIN3 protein turnover was analyzed over time. Compared with that in the control cells, the PLIN3 half-life was considerably decreased in 22RV1 cells stably overexpressing ACSS3, while 22RV1 cells with stable knockout of ACSS3 which were treated with CHX had significantly increased PLIN3 protein half-life levels (Figure 4C). Overall, this suggested that ACSS3 repressed the protein stability of PLIN3 by promoting protein degradation. To further explore which pathway played a major role in this process, a proteasome inhibitor (MG132) and a lysosome inhibitor (chloroquine) were administered to ACSS3‐overexpressing cells. We found that MG132 reversed the decrease in PLIN3 induced by ACSS3 (Figure 4D and Figure S5E), which suggests that ACSS3 represses PLIN3 protein stability mainly by promoting protein degradation mediated by the ubiquitin/proteasomal pathway. Then, ubiquitination‐related immunoprecipitation was used to assess the levels of ubiquitinated PLIN3. The results showed that the level of ubiquitinated PLIN3 was significantly increased in ACSS3‐overexpressing 293T and PCa cells compared to control cells but decreased in prostate cells with stable knockout of ACSS3 (Figure 4E-F and Figure S5F). Collectively, the above results suggested that ACSS3 regulated PLIN3 protein stability by affecting PLIN3 ubiquitination levels.

Previous studies have reported that LD proteins levels are regulated by the ubiquitin-proteasome degradation pathway 49-52. Several ubiquitin E3 ligases have also been implicated in Perilipin family proteins degradation 49-54. Christopher Hooper reported that spartin recruits AIP4 E3 ubiquitin ligase to lipid droplets and by this means regulates the level of ubiquitination of PLIN2 and potentially other lipid-associated proteins 55. These findings led us to expect that AIP4 may be involved in ACSS3 depletion-induced PLIN3 degradation. In 293T cells, AIP4-dependent ubiquitination of PLIN3 was enhanced by the overexpression of AIP4 protein, and ACSS3 expression increased AIP4-mediated PLIN3 ubiquitination. Indeed, the amounts of pulled down AIP4 protein was significantly increased by ACSS3 expression (Figure S5H). Meanwhile, in 22RV1 and C4-2 cells, the amounts of pulled down AIP4 protein was significantly increased by overexpressing ACSS3, while the amounts of pulled down AIP4 protein was significantly decreased by knockout expression ACSS3 (Figure 4E-F). Taken together, these results suggest that ACSS3 recruits AIP4 E3 ubiquitin ligase to lipid droplets and by this means regulates the level of ubiquitination of PLIN3.

### PLIN3 was required for ACSS3-mediated tumor suppression

To confirm PLIN3 function in PCa, we performed GSEA to analyze the gene sets altered by PLIN3 expression in the PCa TCGA dataset. Our results showed that lipid storage-, fatty acid metabolism-, PPAR- and ER Golgi intermediate compartment membrane-related signaling pathways were highly associated with high PLIN3 expression (above the 50^th^ percentile) (Figure S5B). We then examined PLIN3 protein levels by IHC staining in PCa specimens. As shown in Figure S5C and Table S3, PLIN3 protein levels were significantly higher in the PCa tissues (n = 371) than in the normal tissues (n = 107). High PLIN3 expression was negatively correlated with ACSS3 expression and positively correlated with tumor stage and Gleason score.

**Figure 5:**
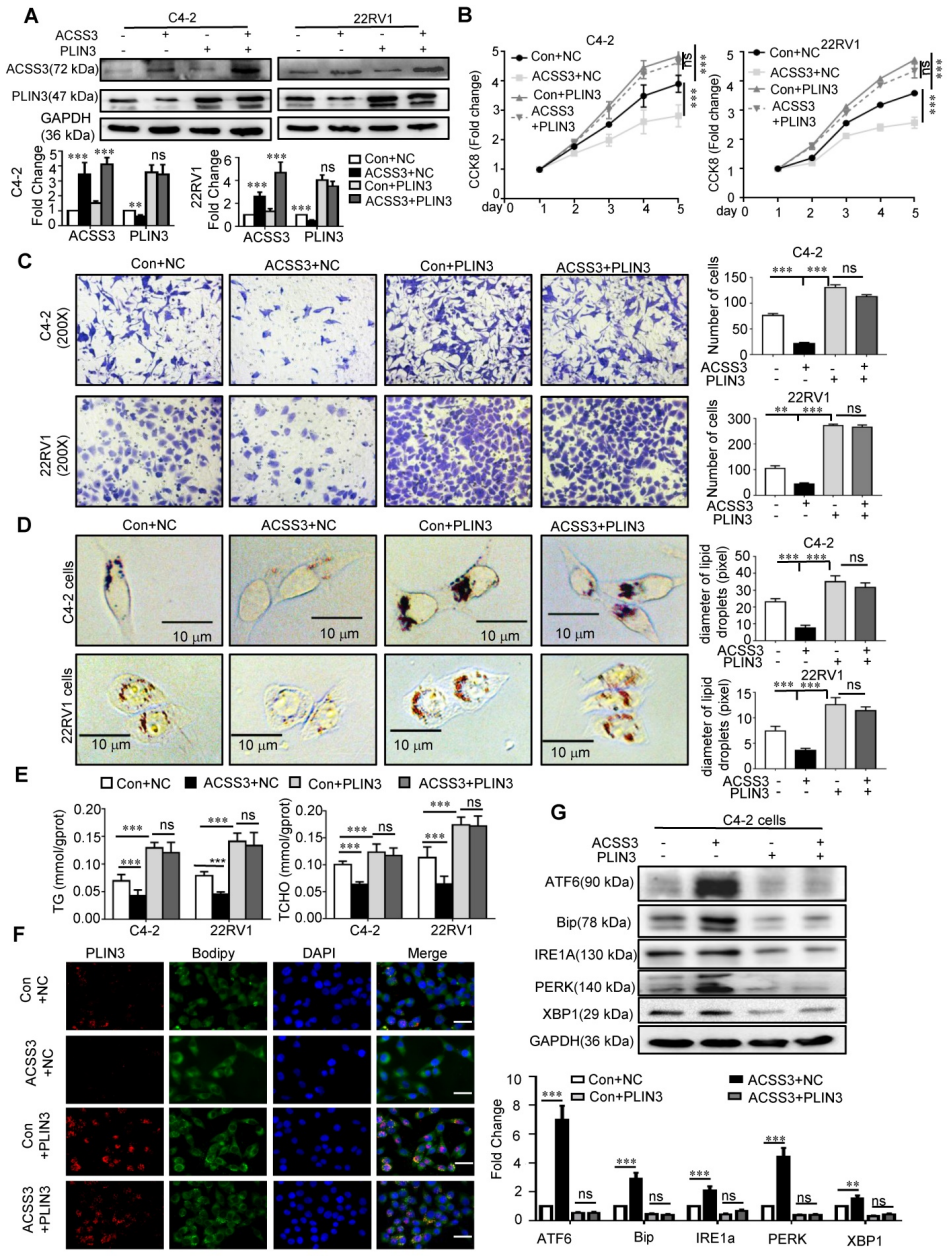
** PLIN3 rescued the ACSS3-mediated tumor cell suppression function.** (A) C4-2 and 22RV1 cells with stable overexpression of ACSS3 and overexpression PLIN3. ACSS3 and PLIN3 protein levels were determined by Western blot analysis. (B) CCK-8 assays were performed to detect PCa cell proliferation. Con is a blank vector control for overexpressed ACSS3, and NC is a blank vector control for overexpressed PLIN3. The graphs show the mean ± SEM (n = 6 per group). (C-E) Migration, oil red staining and TG and cholesterol contents were performed to detect PCa cell migration, lipid accumulation, respectively. The results represent the mean ± SEM from 3 independent experiments. (F) Immunofluorescent was performed to detect the influence of ACSS3 in PLIN3 levels and LDs. The bar is 20 μm. (G) Western blot and densitometry and statistical analysis was performed to detect the protein levels of ER stress markers in C4-2 and 22RV1 cells with stable overexpression of ACSS3 and overexpression PLIN3. The results represent the mean ± SEM from 3 independent experiments. Student's t test; *, *p < 0.05*; **,* p < 0.01*; ***, *p < 0.001*; ns, no significance.

Given that ACSS3 promoted PLIN3 protein degradation, we reasoned that PLIN3 is required for ACSS3-mediated tumor suppression. To test our hypothesis, we stably overexpressed ACSS3 with overexpression of PLIN3 in PCa cells (Figure 5A). CCK-8 assay, migration assay, oil red staining, and Western blotting were performed. Compared with the Con+NC, overexpressed PLIN3 alone or ACSS3+PLIN3 promoted cell proliferation and migration, increased LD deposit size and TG and cholesterol contents. Then compared with overexpressed PLIN3, overexpressed ACSS3+PLIN3 did not affect cell proliferation, migration, LD deposition area, TG and cholesterol content, indicted that PLIN3 rescued the ACSS3-mediated tumor cell suppression function. (Figure 5B-E and Figure 5G). Meanwhile, Immunofluorescent was performed to detect the influence of ACSS3 in PLIN3 levels and LDs and the results indicated that exogenous PLIN3 increases LDs and the PLIN3 coated the LDs, while ACSS3 decrease LDs and the expression of PLIN3 coated the LDs (Figure 5F).

Besides, we successfully identified sgRNA (CRISPR-Cas9) to knock out the expression of PLIN3 in C4-2 and 22RV1 cell lines, and then we overexpressed ACSS3 with simultaneous depletion of PLIN3 in PCa cells (Figure S6A). CCK-8 assay, migration assay, oil red staining, ER Track and Western blotting were performed. The results showed that ACSS3 inhibited cell proliferation and migration, decreased LD deposit size and promoted ER stress, and the ACSS3-mediated tumor cell suppression function was mediated through downregulation of PLIN3 (Figure S6B-G). Similarly, ACSS3 regulated TG and cholesterol contents in tumor cells through PLIN3-dependent signaling (Figure S6D). The observations described above prompted us to investigate the role of ACSS3 *in vivo*. C4-2 cells with stable overexpression of ACSS3 and/or knockout of PLIN3 were implanted subcutaneously or into the tail veins of immunodeficient mice to monitor tumor growth and metastasis. ACSS3 inhibited tumor growth and metastasis in the presence of endogenous PLIN3, while the effects of ACSS3 expression was mediated through downregulation of PLIN3 (Figure S7A-G and Figure S8A). To determine the expression of ACSS3, PLIN3, BIP, PERK in tumor tissues, we conducted IHC staining in tumors (Figure S7B and Figure S8B). TUNEL assays were performed to detect tumor cell apoptosis. Ki67 was used as a surrogate measure for cellular proliferation and apoptosis. BIP and PERK are ER stress markers. As shown in Figure S7B, ACSS3 upregulated the expression of BIP and PERK and downregulated the expression of Ki67 through downregulation of PLIN3. The percentage of apoptotic cells was increased in ACSS3-overexpressing tumors through downregulation of PLIN3. Furthermore, oil red staining was a visual indicator of intracellular lipids in tumor tissues. The results showed that ACSS3 decreased LD deposit size and the effects of ACSS3 expression was mediated through downregulation of PLIN3 (Figure S7C).

Taken together, these results indicated that ACSS3 mediated tumor suppression through PLIN3-dependent signaling.

### ACSS3 inhibited CRPC progression and reversed Enzalutamide resistance

**Figure 6:**
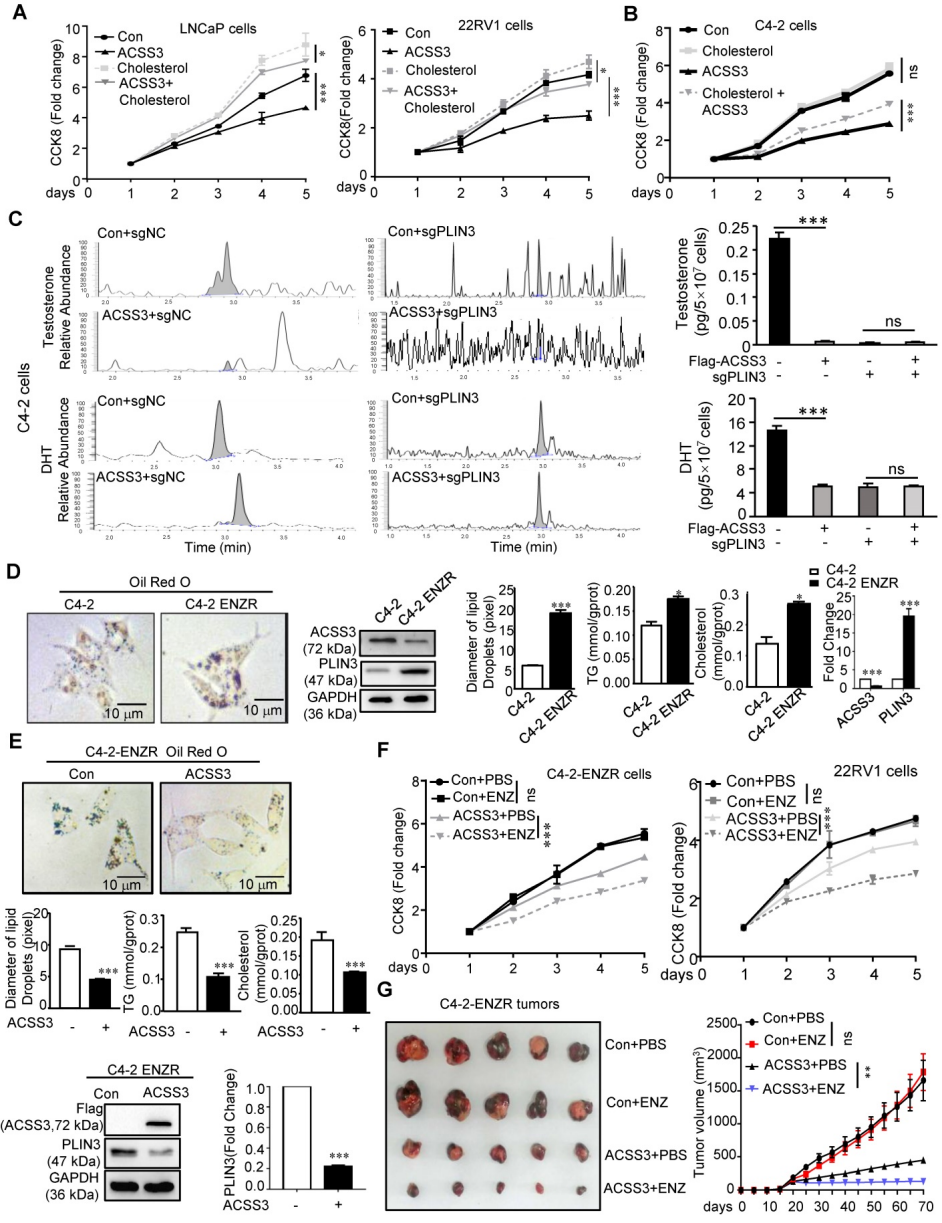
** ACSS3 inhibited CRPC progression and reversed Enzalutamide resistance.** (A-B) LNCaP, 22RV1 and C4-2 cells stably overexpressing ACSS3 were treated with cholesterol (10 µM). A CCK-8 assay was performed. The graphs show the mean ± SEM (n = 6 per group). (C) C4-2 cells with stable overexpression of ACSS3 and PLIN3 knockout. LC-MS was performed to evaluate the T (Testosterone) and DHT (Dihydrotestosterone) contents in the cells. The results represent the mean ± SEM from 3 independent experiments. (D-E) Oil red staining was performed to examine lipid accumulation. Relative diameters of LDs and TG contents and cholesterol contents were measured by image J. The results represent the mean ± SEM from 3 independent experiments*.* Western blot analysis was performed to examine the protein expression of ACSS3 and PLIN3 in C4-2 and C4-2-ENZR (C4-2-enzalutamide resistance), C4-2-ENZR cells stably overexpressing ACSS3 and the control cells. One representative experiment of 3 independent experiments is shown. (F) C4-2-ENZR and 22RV1 cells stably overexpressing ACSS3 were treated with enzalutamide (ENZ). A CCK8 assay was performed. One representative experiment of 3 independent experiments is shown. (G) NCG mice bearing C4-2-ENZR xenografts with stable ACSS3 overexpression were treated with vector control or enzalutamide (10 mg/kg p.o.) for approximately 7 weeks. Tumor volumes were measured. The graphs show the mean ± SEM (n = 6 per group) One-way ANOVA followed by Tukey's multiple comparison test, α = 0.05; *, p < *0.05*; ***, p < *0.001*; ns, no significance.

Cholesterol, the precursor of androgen synthesis, is stored in LDs in the form of cholesterol esters. To investigate if there are other functions of ACSS3 in CRPC progression, LNCaP, 22RV1 (AR-positive and AR-V7-positive cell 59) and C4-2 (derivatives of LNCaP cells and express functional endogenous AR; they can grow in an androgen-independent manner making them an excellent model representing transition of the initial androgen-dependent disease to an androgen-independent state 60) cells stably overexpressing ACSS3 were treated with cholesterol. The results showed that overexpression of ACSS3 inhibited LNCap and 22RV1 cell proliferation, while cholesterol treatment alleviated the ACSS3-mediated suppression (Figure 6A). Interestingly, LNCap and 22RV1 cells (AR positive) responded to cholesterol treatment, while C4-2 cells (CRPC cells) were refractory to cholesterol treatment. And we observed that the overexpression of ACSS3 restored the sensitivity of C4-2 cells to cholesterol treatment (Figure 6B), indicating that ACSS3/PLIN3-mediated proliferation of PCa cell lines could be rescued by cholesterol.

Several researches showed that palmitate caused a dose-dependent increase in lipid accumulation in cells 61-63, and lipid storage promotes endoplasmic reticulum homeostasis 15. To test if palmitate could rescue the phenotype of ACSS3, CCK8 was preformed, and the results showed that palmitate could rescue the inhibitory effect of overexpression of ACSS3 (Figure S9A). Increased intratumoral androgen synthesis is one of the important reasons for the progression of prostate cancer to CRPC, and cholesterol is essential for the synthesis of important androgen. So, we conducted LC/MS to evaluate androgen synthesis in tumor cells, which was the level of testosterone (T) and dihydrotestosterone (DHT) contents in C4-2 cells stably overexpressing ACSS3 and/or knocking down PLIN3. The results showed that restoration of ACSS3 expression significantly decreased the T and DHT contents in C4-2 cells and the effects of ACSS3 expression was mediated through downregulation of PLIN3 (Figure 6C), suggesting that ACSS3 repressed intratumoral androgen synthesis through PLIN3-dependent signaling.

**Figure 7:**
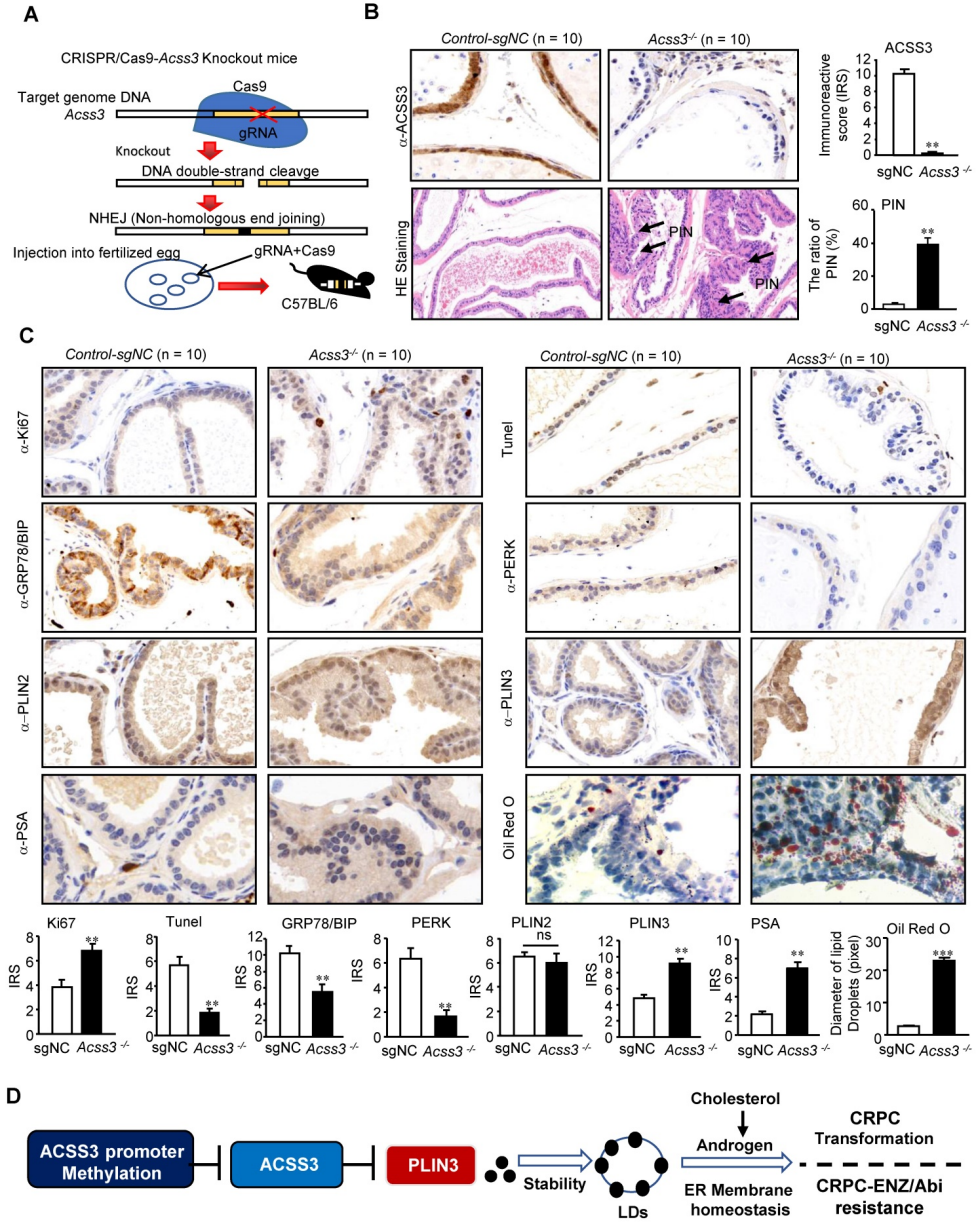
** CRISPR/Cas9-ACSS3 knockout mice confirmed the role of endogenous ACSS3.** (A) CRISPR/Cas9-ACSS3 knockout mice were generated. (B) IHC and HE staining demonstrated that loss of ACSS3 promoted the ratio of prostatic intraepithelial neoplasia (PIN) in the anterior prostates of transgenic mice. The graphs show the mean ± SEM (n = 10 mice per group). (C) IHC staining was performed to detect markers of cellular proliferation, ER stress, apoptosis and AR activity in the anterior prostates of transgenic mice. Oil red staining was performed to detect lipid accumulation in the anterior prostates of transgenic mice. The graphs show the mean ± SEM (n = 10 mice per group), Student's t test; **, p < *0.01*; ***, p < *0.001*; NS, no significance. (D) Proposed model illustrating the protective function of ACSS3/PLIN3-mediated LD elimination in PCa progression.

To investigate whether loss of ACSS3 was involved in Enzalutamide resistance, we used the C4-2-ENZR (C4-2-enzalutamide resistance) cell model. First, oil red staining was performed to detect LD deposits. TG and cholesterol assay kits were used to measure TG and cholesterol contents, and Western blotting was used to measure the expression of ACSS3 and PLIN3 in tumor cells. The results showed that LD deposit size, TG and cholesterol contents and the expression of ACSS3 were reduced in C4-2-ENZR cells, while the expression of PLIN3 was increased in C4-2-ENZR cells compared with that in C4-2 cells (Figure 6D). Overexpression of ACSS3 in C4-2-ENZR cells significantly decreased LD deposits and TG and cholesterol contents (Figure 6E). Subsequently, we overexpressed ACSS3 in C4-2-ENZR cells followed by enzalutamide treatment. The results showed that 22RV1 and C4-2-ENZR cells were refractory to enzalutamide treatment; however, overexpression of ACSS3 restored the sensitivity of 22RV1 and C4-2-ENZR cells to enzalutamide treatment (Figure 6F), indicating that ACSS3 reversed Enzalutamide resistance. Based on the observations described above *in vitro*, we transplanted C4-2-ENZR cells with stable overexpression of ACSS3 into castrated male SCID mice. The mice were treated with enzalutamide (10 mg/kg) twice a week, and similar results were observed. As shown in Figure 6G and Figure S9B, there was no effect of enzalutamide treatment alone on tumor growth and tumor weight; however, ACSS3 overexpression induced the inhibition of tumor growth and tumor weight with enzalutamide treatment, suggesting that ACSS3 can restore enzalutamide treatment sensitivity. Next, the expression of ACSS3, PLIN3 and Ki67 in tumor tissues was detected by IHC staining, and TUNEL assays were performed to detect tumor cell apoptosis. As shown in Figure S9C, there was no effect of enzalutamide treatment alone on Ki67 expression and cell apoptosis; however, ACSS3 overexpression reversed the resistance to enzalutamide. Furthermore, the results of oil red staining showed that restoration of ACSS3 expression in C4-2-ENZR tumors significantly reduced LD deposit size in tumor tissues (Figure S9D). Taken together, these results suggested that ACSS3 repressed CRPC progression, reversed Enzalutamide resistance (Figure 7E).

### CRISPR/Cas9-ACSS3 knockout mice were used to confirm the role of endogenous *Acss3*

To further confirm the role of endogenous ACSS3 *in vivo*, CRISPR/Cas9-*Acss3* knockout mice were generated (Figure 7A). IHC and HE staining demonstrated that loss of ACSS3 increased the ratio of prostatic intraepithelial neoplasia (PIN, a pathological marker of a precancerous lesion) in the anterior prostates of transgenic mice (Figure 7B), suggesting that normal prostatic gland tissue was transforming into tumors due to the absence of ACSS3. Furthermore, consistent with the finding that ACSS3 promoted PLIN3 protein degradation in PCa cell lines, IHC staining showed that the protein level of PLIN3, but not PLIN2, was increased in the *Acss3^-/-^* prostate tissues (Figure 7C-D). Ki67 staining was significantly increased, and BIP and PERK were significantly decreased in the *Acss3^-/-^* prostate, suggesting that ACSS3 inhibited cell proliferation and promoted ER stress. TUNEL staining was conducted to assess the effect of endogenous ACSS3 on cellular survival *in vivo*. The percentage of apoptotic cells was reduced in the *Acss3^-/-^* prostate tissues compared to the control prostate tissues (Figure 7C-D). Subsequently, TG and cholesterol contents were measured as quantitative indicators of lipid accumulation in prostate tissues. The results showed that TG and cholesterol contents were increased in the *Acss3^-/-^* prostate tissues (Figure S10A-B). The weight of *Acss3^-/-^* and control mouse was measured. The result indicated that KO Acss3 increased slightly increase the body weight (Figure S10C). Next, we conducted PSA staining to measure androgen receptor (AR) activity. PSA was significantly increased in the *Acss3^-/-^* prostate compared to the control prostate (Figure 7C-D), suggesting that ACSS3/PLIN3-mediated LD elimination reduced cholesterol content in prostate cells, thus downregulating androgen synthesis, thereby repressing AR activity. Furthermore, oil red staining confirmed that LD deposits were obviously increased in the *Acss3^-/-^* prostate (Figure 7C-D).

## Discussion

Since Charles Haggins first demonstrated in 1940 that blocking hormone production inhibited PCa progression, endocrine therapy has been the first line of treatment for advanced PCa. Endocrine therapy for PCa blocks the androgen/AR signaling pathway 19-21. However, this continuous “blocking” does not prevent tumor progression. Although androgens from testicular and adrenal glands are blocked, intratumoral androgen synthesis is increased 12-14, 22). AR variants are more sensitive to low levels of androgen and drive disease progression 23. PCa progresses to CRPC, which is treated with Enzalutamide to further inhibit AR activity or key enzymes of androgen synthesis, but tumors still progress and become incurable. Investigation of new therapeutic perspectives is urgently needed 24-26. The latest studies have demonstrated that AR variation-mediated lipid/cholesterol deposits drive CRPC progression. Lipid/cholesterol, a precursor of steroid synthesis, increases the production of intratumoral androgens and is highly upregulated in Enzalutamide-resistant CRPC cells 11-14, 28-27. In the current study, we found that ACSS3/PLIN3-mediated LD elimination significantly reduced lipid/cholesterol contents in tumor cells, thus repressing intratumoral androgen synthesis, thereby inhibiting CRPC progression and Enzalutamide resistance.

Epidemiological studies suggest that high-fat diets play important roles in PCa progression 28-32. A high-fat diet provides materials for fat cells to promote the synthesis and storage of LDs 33, and abnormal accumulation of LDs in fat cells eventually leads to obesity. Obesity requires lipid reduction. Tumor cells with abnormal lipid metabolism also need to reduce the accumulation of LDs. LDs are mainly composed of neutral fats, including triglycerides and cholesterol esters, which are coated with a single layer of phospholipid molecules and various proteins. Perilipin family proteins are the predominant LD-associated proteins 16-18. Some studies have indicated that LD deposits can stabilize the ER, relieve ER stress, and enhance tumor cell viability 15, 34-37. Here, we demonstrated that restoration of ACSS3 expression in PCa cells significantly reduced LD deposits, which increased ER stress-mediated apoptosis, thus inhibiting tumor growth and metastasis. We also used PC3 cells to detect the effect of ACSS3 in AR negative PCa cells, and the results showed that ACSS3 inhibited the proliferation of PC3 cells. Previous studies demonstrated that the accumulation of lipid droplet could maintain the endoplasmic reticulum stability, which promotes tumor cell viability and proliferation 15. These results indicated that ACSS3 overexpression inhibit proliferation of PC3 cells independent on regulating androgens. Some studies have reported that Perilipin family proteins are degraded by the ubiquitin-proteasome degradation pathway 48, 54-55 and other studies showed that LD-associated proteins perilipin are CMA substrates and their degradation through CMA precedes lipolysis 56. In this study, we found that ACSS3 recruits AIP4 E3 ubiquitin ligase to lipid droplets and by this means regulates the level of ubiquitination of PLIN3. Besides, ACSS3 inhibits CRPC progression and reverses Enzalutamide resistance through reducing intratumoral LD deposits.

## Materials and methods

### Cell culture and reagents

Human PCa cell lines DU145, PC3, C4-2, LNCAP, 22RV1 were purchased from the American Type Culture Collection (ATCC). All PCa cells were cultured in RPMI 1640 medium supplemented with 10% Fetal bovine serum (FBS) at 37 °C in 5% CO_2_. 293T cells were cultured in DMEM medium supplemented with 10% Fetal bovine serum (FBS) (Gibco) at 37 °C in 5% CO_2_. RWPE-1 cells were cultured in K-SMF medium (Gibco) at 37 °C in 5% CO_2_.

Expression lentivirus for ACSS3, PLIN3, sgRNA for ACSS3 and PLIN3, corresponding control vector were all purchased from Genechem. All steps were executed according to the manufacturer's instructed protocol. Human ACSS3 (NM_024560) cDNA was amplified with PCR and cloned into a GV492 lentiviral vector (GeneChem, Shanghai, China). Human PLIN3 (BC007566) cDNA was amplified with PCR and cloned into a GV382 lentiviral vector (GeneChem, Shanghai, China). Oligos of ACCS3/PLIN3 sgRNAs were synthesized and inserted into a GV392 (U6-sgRNA-EF1a-Cas9-FLAG-P2A-puro) lentiviral vector (GeneChem, Shanghai, China): sgACSS3-1: TAGTCGCATTGATCATGTAA, sgACSS3-2: CTCCCGGTCGTGACCTTGAT, sgControl: CGCTTCCGCGGCCCGTTCAA; sgPLIN3-1: ACAGAGCTACTTCGTACGTC, sgPLIN3-2: GCACGCCTATGAGCACTCGC, sgControl: CGCTTCCGCGGCCCGTTCAA. Stable cell lines that expressed ACSS3, PLIN3. sgRNA-ACSS3 (sgACSS3), sgRNA-PLIN3 (sgPLIN3) were selected for 15 days with 2 ug/ml puromycin. Expression plasmids for Flag-ACSS3, His-PLIN3 and Myc-Ubi were also obtained from Genechem. For transfection, 293T cells were cultured in 6-well plants and transfected with plasmids using Lipofectamine 2000 reagents (Thermo Fisher Scientific, Waltham), according to the manufacturer's instructed protocol. Cholesterol, DNA methyltransferase inhibitor 5-AZA, protein synthesis inhibitor cycloheximide, proteasome inhibitor MG132, lysosome inhibitor chloroquine and Enzalutamide (ENZ) were all purchased from MedChemExpress.

### Human samples

Paired cancer and adjacent noncancer paraffin tissue sections for IHC staining were purchased from Xi'an alenabio (PR1921b), shanghai Outdo Biotech (HProA150CS01), Superbiotek (PRC1021), Suzhou CCELL (20190522R80), Xi'an ZK bioaitech (M821601). Information including pathology diagnosis and clinical stage were directly provided by companies. The samples for BSP are fresh prostate cancer tissues with matched adjacent normal tissues, which were collected from Huazhong University of Science and Technology affiliated Union Hospital and stored at - 80 °C. For the use of these clinical materials for research purposes, prior patients' consents and approval from the Institutional Research Ethics Committee were obtained.

### RNA Sequencing

The RNA sequencing was performed by OEbiotech. Briefly, the collected cells were processed for RNA isolation using TRIzol reagent (Invitrogen) according to manufacturer's protocol. To the constructed RNA library, remove the rRNA using the TruSeq Stranded Total RNA with Ribo-Zero Gold kit, and reverse transcription was performed for cDNA. The purified DNA was amplified by PCR., followed by qualitied inspection with Agilent Bioanalyzer 2100 system (Agilent Technologies, USA), and then Illumina sequencer was used for sequencing. The differentially expressed mRNA were further analyzed based on |Fold Change| > 1.5 and p value < 0.05. The Database for Annotation, Visualization and Integrated Discovery (DAVID, https://david.ncifcrf.gov/) was used to perform Gene Ontology (GO) functional and KEGG pathway enrichment analyses, considered p < 0.05 as statistically significant. The microarray data generated in this paper is available in GSA-Human (https://bigd.big.ac.cn/gsa-human/), under the Bioproject (PRJCA003107), accession HRA000240.

### LC/MS analysis

All experiments were performed as described previously 38-39. Briefly, the collected cells were lysed and stored at -80 °C until LC-MS analysis. ACQUITY UPLC I-Class system (Waters Corporation, Milford) coupled with VION IMS QTOF Mass spectrometer (Waters Corporation, Milford) was used to analyze the metabolic profiling in both ESI positive and ESI negative ion modes. An ACQUITY UPLC BEH C18 column (1.7 µm, 2.1 * 100 mm) were employed in both positive and negative modes. Water and Acetonitrile/Methanol 2/3 (v/v), both containing 0.1% formic acid were used as mobile phases A and B, respectively. Linear gradient: 0 min, 1% B; 1 min, 30% B; 2.5 min, 60% B; 6.5 min, 90% B; 8.5 min, 100% B; 10.7 min, 100% B; 10.8 min, 1% B and 13 min, 1%B. The flow rate was 0.4 mL/min and column temperature was 45 °C. All the samples were kept at 4 °C during the analysis. The injection volume was 1 µL. The QCs were injected at regular intervals (every 10 samples) throughout the analytical run to provide a set of data from which repeatability can be assessed. The differential metabolites were selected on the basis of the combination of a statistically significant threshold of variable influence on projection (VIP) values obtained from the OPLS-DA model and p values from a two-tailed Student's t test on the normalized peak areas, where metabolites with VIP values larger than 1.0 and p values less than 0.05 were considered as differential metabolites. Metabolites were identified by progenesis QI (Waters Corporation, Milford) Data Processing Software, based on public databases such as http://www.hmdb.ca/; http://www.lipidmaps.org/ and self-built databases. The LC/MS lipidomic date generated in this paper is available in Metabolights (http://www.ebi.ac.uk/metabolights/), study identifier MTBLS1948.

### RNA isolation and real-time PCR analysis

Total RNA from cells was extracted using the TRizol reagent (Thermo, Massachusetts) according to the manufacturer's instructions. The purity and concentration of the RNA solution were detected by the NanoDrop 2000 spectrophotometer (NanoDrop Technologies, Wilmington). Using 0.5 ug of cell RNAs, reverse transcription was performed according to the instructions of the RevertAid First Strand cDNA Synthesis kit (TaKaRa). Real-time quantitative PCR was performed with the SYBR Green mix (TaKaRa) on a StepOne Plus real-time PCR system (Life Technologies, Carlsbad, CA). Genes primers ACSS3 (forward, 5'-CCGGTCGTGACCTTGATTGG-3', reverse, 5'-CGTTGTGCCAGATGTGTAAAGA-3'), PLIN3 (forward, 5'- TATGCCTCCACCAAGGAGAG-3', reverse, 5'-ATTCGCTGGCTGATGCAATCT-3'), PLIN2 (forward, 5'-ATGGCATCCGTTGCAGTTGAT-3', reverse, 5'-GGACATGAGGTCATACGTGGAG-3'), PERK (forward, 5'-GGAAACGAGAGCCGGATTTATT-3', reverse, 5'-ACTATGTCCATTATGGCAGCTTC-3'), BIP (forward, 5'-GAAAGAAGGTTACCCATGCAGT-3', reverse, 5'-CAGGCCATAAGCAATAGCAGC-3'), IRE1A (forward, 5'-CACAGTGACGCTTCCTGAAAC-3', reverse, 5'-GCCATCATTAGGATCTGGGAGA-3'), XBP1 (forward, 5'-CCCTCCAGAACATCTCCCCAT-3', reverse, 5'-ACATGACTGGGTCCAAGTTGT-3'), CHOP (forward, 5'-GGAAACAGAGTGGTCATTCCC-3', reverse, 5'-CTGCTTGAGCCGTTCATTCTC-3'), ATF6 (forward, 5'-TCCTCGGTCAGTGGACTCTTA-3', reverse, 5'-CTTGGGCTGAATTGAAGGTTTTG-3'), HERK (forward, 5'-ATGGAGTCCGAGACCGAAC-3', reverse, 5'-TTGGTGATCCAACAACAGCTT-3'), EDEM1 (forward, 5'- GCTACGACAACTACATGGCTC-3', reverse, 5'-GACTTGGACGGTGGAATCTTT-3'), GAPDH (forward, 5'-TCAAGAAGGTGGTGAAGCAG-3', reverse, 5'-CGTCAAAGGTGGAGGAGTG-3') were bought from Sangon Biotech.

### Bisulfite genomic Sequence Polymerase Chain Reaction (PCR) (BSP)

BSP was carried out according to standard methods as described previously 40. In brief, sequences of 2,000 bp from upstream of the transcription initiation site to downstream of the transcription initiation site in promoter areas were download from NCBI. The bioinformatics predictions of promoter-related CpG islands were analyzed by the UCSC Genome Browser on the Human Dec 2013 assembly (hg38). Genomic DNAs were treated with sodium bisulfite, and PCR was carried out using the primers (ACSS3-Forward: 5'-TTGTTGGTTTTTTGTAGGTTAGAGG-3', ACSS3-Reverse: 5'-CCAAAAATTTAAAAACCACACTAAAATC-3') which were designed on the online MethPrimer software (http://www.urogene.org/methprimer/). Finally, Bisulfite-sequencing analysis was performed with ABI3730XL sequencing machine.

### Methylation-Specific Polymerase Chain Reaction (PCR) (MSP)

Genomic DNA from PCa cell lines and prostate epithelial cell (RWPE-1) were prepared with Cell Genomic DNA Extraction Kit (ELK Biotechnology Cat. EP007) according to the manufacturer's instructed protocol. Genomic DNAs were treated with sodium bisulfite using the Methylation-Gold Kit. PCR was carried out using the primers which were designed amplify the regions of interest. 10 µl of the production with Dye was subjected to agarose gel at 100 V for 30 min. Observe and photograph under UV lamp.

### Co-immunoprecipitation (CO-IP)

CO-IP were done as described previously 41. In brief, the cells were cultured in T75-culture bottle, and washed by cold PBS, followed by lysed using Triton-lysis buffer. After centrifuged at 12,000×g for 10 min at 4 °C, per 100 µL of the supernatant added with 20 µL Protein A/G PLUS-Agarose (Santa Cruz, CA, USA) and incubated with the appropriate antibody overnight at 4 °C. The next day, the immunoprecipitants were washed three times and boiled with 2×SDS Loading Buffer. Do western blotting assay according to the protocol above.

### Immunohistochemical staining assay

The paraffin-embedded specimens were cut into 4 µm thick section for immunohistochemical staining. Sections were deparaffinized with xylenes and rehydrated, incubated in EDTA at 120 °C for 5 min for antigen retrieval. Treated with 3% H_2_O_2_ in methanol for 15 min to quench the endogenous peroxidase activity, the sections were then incubated with fetal bovine serum for 1 h to block the nonspecific binding. And then, the tissue sections were incubated with primary antibodies overnight at 4 °C. The primary antibodies include: ACSS3 (1:200, ab188009 Abcam, USA), PLIN3 (1:500, 10694-1-AP, Proteintech, China), PLIN2 (1:500, ab108323, Abcam, USA), PERK (1:200, 3192S, CST, USA), GRP78/BIP (1:200, 3177, CST, USA), Ki67(1:10000, 27309-1-AP, Proteintech, China), PSA (1:200, 10679-1-AP, Proteintech, China). The slides were washed three times with PBS, followed by incubated with a biotinylated secondary antibody (Proteintech, China). Finally, 3, 3-diaminobenzidine tetrahydrochloride (DAB) was used for visualizing the immune complexes, followed by counterstain with hematoxylin. According to the immunoreactive score (IRS), analyze the expression of protein in tissue sections. IRS scores of 0-1 indicate negative; scores of 2-3 indicate mild; scores of 4-8 indicate moderate; scores of 9-12 indicate strongly positive.

### Western blotting

The cells were harvested and lysed with RIPA Lysis Buffer which added protease inhibitor cocktail and PMSF freshly. Protein concentration was detected using BSA kit. 30 µg of protein was subjected to SDS-PAGE gel. Separated by gel electrophoresis, the proteins were then transferred to polyvinylidene difluoride (PVDF) membrane. The membranes were blocked with 5% non-fat dried skimmed milk for 2 h at room temperature, and then incubated with corresponding primary antibodies overnight in 4 °C. The primary antibodies include: ACSS3 (1:1000, ab188009 Abcam, USA), PLIN3 (1:5000, 10694-1-AP, Proteintech, China), PLIN2 (1:2000, ab108323, Abcam, USA), IRE1-alpha (1:1000, 3294S, CST, USA), PERK (1:1000, 3192S, CST, USA), Bip (1:1000, 3177, CST, USA), ATF6 (1:1000, 65880S, CST, USA), XBP1 (1:1000, ab37152, Abcam, USA), CHOP (1:1000, CST, USA), PARPs (1:2000, 9532, CST, USA), cleaved Caspase 3 (1:1000, 9661S, CST, USA), IRE1-alpha [p-Ser724] (1:1000, NB100-2323, NOVUS, USA), AIP4(1:1000, 20920-1-AP, Proteintech, China), GAPDH (1:3000, 10494-1-AP, Proteintech, China), β-Actin (1:3000, 20536-1-AP, Proteintech, China). The membranes were detected using horseradish peroxidase-conjugated secondary antibodies (1:3000, SA00001-2, Proteintech, China) followed by exposure to enhanced chemiluminescence substrate (Thermo, Massachusetts, USA).

### ER tracker staining assay

Cells plated in petri dish special for confocal microscope at triplicate, were washed twice using Hanks' Balanced Salt Solution with Ca^2+^ & Mg^2+^. And then, the cells were incubated for 15 min with preheated working fluid in ER-Tracker Red Kit (Beyotime). The cells were then washed twice with RPMI 1640 medium and fixed in 4% paraformaldehyde for 5 min. Adding DAPI is to stain Nuclei. Images were acquired under a confocal microscope.

### Cell viability assay

The cell proliferation was tested by using the Cell Counting Kit-8 (CCK8) assay according to the manufacturer's protocol. In brief, 3000 cells per well were plated into a 96-well plate and cultured at 37 °C in 5% CO_2_. Per well was added with 10ul of CCK8 reagent (DOJINDO Laboratories, Kumamoto, Japan), and incubated for 2 h at 37 °C, followed by measured the optical density at 450 nm using a spectrometer. Cell viability was calculated after 24 h, 48 h, 72 h and 96 h according to the relative optical density. All experiments were performed in triplicate.

### Transwell assay

To evaluate the ability of cell migration, the transwell assays were preformed using 24-well transwell chambers with 8 µm pore membranes (Corning LifeSciences). Incubated in serum-free medium for 24 h, 20,000 cells were seeded into in the top chamber of the insert, by followed adding media containing 10% FBS in the lower chamber as a chemoattractant. The chambers were cultured at 37 °C for 48 h, washed twice with PBS, and fixed in 100% methanol on the ice for 15 min. Then the cells were stained with 0.05% crystal violet for 15 mins, followed by carefully cleaning away the cells in the top chamber of the insert. Chose 5 fields randomly and count for analyzing. All experiments were performed in triplicate.

To evaluate the ability of cell invasion, the transwell assays were preformed using 24-well transwell chambers with 8 µm pore membranes (Corning LifeSciences). The transwell chambers for invasion assays must be pre-coated with Matrigel (BD Biosciences, San Jose, CA, USA). The next steps are the same as above.

### Oil Red O staining assay

The working oil red O solution was obtained by diluting saturated oil red O solution (Servicebio technology) at 6:4 with distilled water. For frozen tissue section, optimal cutting temperature (OCT)-embedded tissue was cut to 8 µm sections and fixed in 4% paraformaldehyde for 10 min before staining. The cells which were cultured in 6-well plate were washed twice using PBS and fixed in 4% paraformaldehyde for 10 min before staining. The cells and tissue sections were incubated in 60% isopropanol for 30 s, and then incubated in oil red O staining solution avoiding light for 10 min at room temperature. The tissue sections needed to be counterstained with hematoxylin for 2 min. Finally, washed twice, photographed and counted. Using image J to measure the length of lipid droplets in 100 cells and the length of lipid droplets was measured in pixels.

### TG and cholesterol Measurement Assay

TG or cholesterol content in cells and serum was measured using the TG assay kit (cat. A110-1-1, Bioengineering) or Total cholesterol assay kit (cat. A111-1-1, Bioengineering), according to the manufacturer's instructed protocol. In brief, the cells or tissues were lysed in normal saline infusion with ultrasonic apparatus, and serum was prepared by centrifuged at 12,000 × g for 10 min at 4 °C. 2.5 µl of reagent in the kit was added into 250 µl of specimen in a 96-well plate, and was cultured avoiding light at 37 °C for 5 min. Finally, the optical density was measured at 510 nm using a spectrometer. Protein concentration as a control was detected using BSA kit. All experiments were performed in triplicate.

### TUNEL assays

TUNEL assays were performed with colorimetric TUNEL apoptosis assay kit according to the manufacturer's instructions (Beyotime, China). In brief, the sample sections were incubated in the TUNEL reaction mixture. The sections were rinsed and visualized using DAB. The number of TUNEL-positive cells was counted in six fields randomly, and the apoptosis index for each field was calculated as the percent of TUNEL-positive cells relative to the total cells.

### Transmission electron microscopy (TEM)

Briefly, the cells were washed using cold PBS softly and fastly, and fixed with 2.5% phosphate-buffered glutaraldehyde (Servicebio) overnight at 4 °C. After subsequent buffer washes, the samples were post-fixed in 1% phosphate-buffered osmium tetroxide for 2 h at room temperature. The samples were then infiltrated and embedded in EMbed-812 (Electron Microscopy Sciences), followed by sectioned and double stained with uranyl acetate and lead citrate. Finally, the specimens were scanned and examined with an H-7650 transmission electron microscope (Hitachi).

### *Acss3^-/-^* mouse Construction

The *Acss3* knock-out mice was constructed by the CRISP/Cas9 technique. In vitro, the sgRNAs of Acss3 were predicted according to the exons of *Acss3*, and the RNA sequencing was carried out to searching for the most effective sgRNA: sgAcss3: TGATCGGCACATTGAAAATGG. The *Acss3*-sgRNA was transcribed into RNA in vitro, and then microinjected into mouse zygote with Cas9 mRNA. After 2 weeks, the mice were sequenced, and their genotypes were analyzed and identified to obtain the Founder mouse with gene mutation. The Founder mice were selected to mate with wild-type C57 mice, and the genotype of the born mice was identified two weeks late to establish the F1 generation mice (*Acss3^-/+^*). According to the results of F1 generation identification, the F1 mice were selected to mate with each other, and the genotypes of the born mice were identified two weeks later to gain F2 generation mice (*Acss3^-/-^*).

### Tumor xenografts

Subcutaneous xenograft model and Nude mouse tail vein metastasis model experiments were approved by the Animal Care and Use Committee of Tongji Medical College of Huazhong University of Science and Technology. Male BALB/c nude mice (4-5 weeks old), which were surgically castrated, were purchased from the Vital River Laboratory Animal Technology Co. Ltd. (Beijing, China), and randomized five into a group. For tumor formation assay, 2×10^6^ cells in a 1:1 mixture of PBS and Matrigel (BD, USA) were inoculated subcutaneously into each mouse. Tumor size was measured every 3 days. The mice were euthanized after 20 days, and the tumors were excised, measured and stored for future study. For tumor metastasis model, 1×10^6^ cells in PBS were injected into each mouse. After 45 days, whole body luminescence imaging of mice using IVIS-Spectrum optical imager was obtained after anesthesia. Then the mice were euthanized and lung was taken out and embedded with paraffin for future study. Male NCG mice (4-5 weeks old) were purchased from the GemPharmatech., Ltd. (Nanjing, China), which were surgically castrated for subcutaneous xenograft model experiments about detesting ACSS3 reversed Enzalutamide resistance in vitro.

### Bioinformatics Analysis

The mRNA expression of genes in PCa patients, adjacent normal tissues and clinical data of patients in TCGA-PRAD Database were obtained from the cBioPortal (http://www.cbioportal.org/public-porta). The gene expression profiling datasets (Taylor GSE21034, Tomlins GSE6099, Lapointe GSE3933, Grasso) were obtained from Gene Expression Omnibus (GEO; https://www.ncbi.nlm.nih.gov/geo/) database. The independent lipid metabolism-related gene sets were directly downloaded from oncomine database (https://www.oncomine.org). We have added the gene list in the Table S1. The gene set enrichment analysis (GSEA) was used to assess pathways enriched in the gene set that based on false discovery rate (FDR) < 0.25 and nominal p < 0.05.

### Statistical analysis

All the experiments were performed in triplicate and statistical analysis was done with GraphPad Prism 5. Normally distributed continuous variables are represented as mean ± SEM, while non-normally distributed continuous variables are expressed as median. For equivalent variables with a normal distribution, Student's t-test was applied to compare the two experimental groups. Mann-Whitney U test was used to compare non-normal distributional variables between two groups. One-way ANOVA followed by Tukey's multiple comparison test was used to compare multiple groups. Pearson correlation test was used to assess the correlation between variable with normal distribution, while Spearman correlation test was used to assess the correlation between variable with non-normal distribution. Survival rate was plotted using Kaplan-Meier method and analyzed using the log-rank test. A Cox proportional hazard regression model was performed for the multivariate analysis of the combinatorial contribution of ACSS3 and the clinicopathological features to the survival of the patients. p values of < 0.05 were considered statistically significant.

## Supplementary Material

Supplementary figures and tables.
