# Supplementary material for: ACSS3 represses prostate cancer progression through downregulating lipid droplet-associated protein PLIN3
Source: Theranostics. 2021 Jan 1;11(2):841–60. doi: 10.7150/thno.49384 (PMC7738848; doi:10.7150/thno.49384)
Supplement: Supplementary file 1 — Supplementary figures and tables. [file thnov11p0841s1.pdf]

Table S1. Lipid-metabolism gene list (147, GO Biological Process) in datasets

	TCGA		Grasso		Lapointe		Tomils		Taylor	
gene-list	FC	p vaule	FC	p vaule	FC	p vaule	FC	p vaule	FC	p vaule
AACS	0.30852	0.00033	1.21	0.0001	1.22	4E-04	-1.06	0.382	1.06	0.03
AASDH	-0.1224	0.18276	-1.03	0.407	NA	NA	NA	NA	1.04	0.254
ABCA2	0.56436	2.8E-08	1.14	0.023	NA	NA	NA	NA	1.05	0.041
ABCB4	-0.164	0.22939	-1.14	0.228	NA	NA	1.44	0.005	1.01	0.352
ACAD8	0.18436	0.22705	1.74	5E-05	1.16	0.097	1.11	0.321	1.33	0.0008
ACOT2	-0.2182	0.03009	-1.35	9E-05	NA	NA	NA	NA	-1.21	2E-07
ACOT4	-0.2929	0.01409	1.02	0.6	-1.09	0.022	-1.17	0.189	1	0.408
ACOT7	0.19452	0.01714	-1.18	0.018	-1.41	2E-06	1.09	0.636	-1.04	0.108
ACOT8	0.43015	1.2E-10	1.26	2E-05	NA	NA	-1.36	0.024	-1	0.491
ACOT9	-0.2423	0.00076	-1.06	0.144	NA	NA	NA	NA	-1.17	0.0003
ACOX1	0.01908	0.80042	1.1	0.914	1.13	0.025	1.33	0.116	1.04	0.094
ACSBG2	0.2482	0.15375	1.33	0.021	NA	NA	NA	NA	1.04	0.069
ACSF2	-1.5662	6.4E-20	-1.83	3E-09	-2.01	1E-17	-2.37	7E-07	-1.57	6E-16
ACSF3	0.14459	0.04041	-1.09	0.143	NA	NA	NA	NA	-1.02	0.1
ACSL3	0.38547	0.00762	NA	NA	1.5	2E-06	-1.42	0.175	1.42	0.0006
ACSL4	-1.2238	4.5E-16	-1.49	2E-05	-1.28	3E-05	NA	NA	-1.45	7E-07
ACSL6	-1.5158	1.5E-15	-1.19	0.143	NA	NA	1.47	0.065	1.05	0.088
ACSM1	2.67189	1.3E-16	4.4	3E-09	NA	NA	3.91	1E-04	2.74	2E-19
ACSM5	-0.7561	1.6E-06	-1.69	0.276	NA	NA	NA	NA	1.08	0.015
ACSS3	-1.5041	5.2E-18	-1.57	2E-06	-1.73	3E-14	-4.93	7E-08	-1.52	2E-10
ADIPOR1	0.25528	0.00038	1.38	3E-09	1.23	6E-06	1.9	9E-05	1.14	0.002
ADIPOR2	-0.1899	0.00608	-1.06	0.062	1.04	0.273	1.07	0.659	-1.06	0.088
AGPS	-0.9131	3.4E-12	-1.67	0.0001	-1.51	2E-08	1.18	0.118	-1.17	0.003
AKR1C2	-1.3336	4.5E-11	NA	NA	NA	NA	NA	NA	-1.2	0.002
AKR1C3	-0.1532	0.42054	1.04	0.63	NA	NA	1.85	0.016		
ALDH1A3	1.06698	5.3E-11	1.85	6E-05	1.35	0.006	2.14	0.026	1.52	2E-07
ALDH3A2	-0.4567	5.4E-06	-1.6	2E-07	-1.52	3E-08	-1.43	0.083	-1.23	0.0002
ALDH3B1	-0.715	5E-09	-1.02	0.618	-1.06	0.275	1.31	0.048	1.05	0.013
ALDH3B2	1.05018	9.2E-06	1.43	5E-05	NA	NA	-1.68	0.052	1.31	2E-12
ALOX12B	0.4525	0.04235	1.05	0.661	NA	NA	NA	NA	-1	0.449
ALOX15	1.81854	1.5E-05	1.41	0.013	NA	NA	NA	NA	1.12	2E-07
ALOX15B	-0.2357	0.60716	-1.02	0.382	-1.54	1E-04	NA	NA	1.04	0.371
ANXA1	-1.1716	6.4E-13	-1.33	0.009	-2.13	2E-10	-15.2	1E-10	-1.29	0.014
AOAH	-0.113	0.50813	-1.03	0.407	NA	NA	-1.66	0.004	1.11	0.049
APOA4	1.04756	0.12089	-1.01	0.476	NA	NA	NA	NA	1.04	0.157
APOB48R	#N/A	#N/A	1.2	0.051	NA	NA	NA	NA	1.04	0.188
APOBEC1	0.69774	0.12756	1.45	0.738	NA	NA	NA	NA	1.07	0.032
APOC1	2.07012	1.1E-23	2.87	2E-06	1.89	5E-06	2.34	9E-05	1.24	0.0001
APOC4	0.48042	0.03226	1.68	4E-09	NA	NA	-1.48	0.012	1.04	0.069
APOD	0.63833	0.00056	1.38	0.007	NA	NA	2.37	0.001	1.22	0.019
APOF	2.52724	7.9E-18	2.12	0.0001	NA	NA	NA	NA	1.38	2E-12
APOL1	-0.7622	4.3E-07	-1.39	0.002	NA	NA	NA	NA	1.02	0.244
APOL2	0.21944	0.00292	-1.33	0.0005	NA	NA	NA	NA	1.03	0.079
APOL5	-0.1462	0.46117	-1.08	0.308	NA	NA	NA	NA	1.07	0.037
ATP8B1	-0.3894	0.00222	1	0.514	1.02	0.405	1.04	0.552	-1.04	0.323
BAAT	0.41377	0.10697	1.43	0.019	1.07	0.169	1.23	0.116	1.04	0.123
BTN2A1	-0.5107	4.9E-10	-1.27	0.005	-1.34	5E-07	-1.14	0.222	-1.16	3E-09
BTN3A1	-0.3201	0.00652	-1.38	0.009	-1.28	0.002	-2.19	2E-05	-1	0.48
BTNL3	0.06298	0.75045	-1.01	0.489	NA	NA	NA	NA	1.03	0.244
C10orf129	NA	NA	NA	NA	NA	NA	NA	NA	1.08	0.002
CD36	-1.0597	5E-07	-1.4	0.256	NA	NA	-1.49	0.002	1.02	0.321
CDS1	0.22717	0.02099	1.25	0.926	1.25	0.002	1.72	0.01	1.17	0.002
CEPT1	-0.216	0.04854	1.11	0.917	1.06	0.147	1.59	0.001	1.03	0.251
CETP	0.63069	4.9E-05	1.23	0.84	NA	NA	1.08	0.29	1.07	0.016
CH25H	-0.7746	0.00073	1	0.504	-1.02	0.412	NA	NA	1.19	0.007

CHKA	0.23426	0.00466	1.5	6E-05	1.37	2E-06	1.21	0.139	1.09	0.047
CLU	-2.0822	2.1E-32	-2.09	6E-07	-2.44	2E-19	-2.27	4E-04	-1.9	3E-11
CORIN	-0.6438	0.00209	-1.18	0.21	-1.1	0.076	-1.2	0.18	-1.09	0.031
CPNE1	0.04914	0.53947	1.05	0.758	1.4	5E-09	-1.01	0.463	-1.15	0.0009
CPNE3	0.06382	0.54573	1.33	5E-07	1.12	0.057	-1.14	0.211	1.13	0.027
CPNE6	-2.9879	4E-18	-2.43	2E-11	NA	NA	1.04	0.662	-1.22	5E-06
CPNE7	1.91866	1.4E-15	2.51	1E-12	NA	NA	NA	NA	1.19	4E-06
CYP27B1	0.73144	2.8E-07	1.41	0.002	NA	NA	NA	NA	1.09	0.034
DAGLA	-0.4866	0.00092	-1.97	0.072	NA	NA	-1.82	3E-04	-1.03	0.137
DAGLB	0.24235	1.4E-05	-1.08	0.16	NA	NA	-1.64	5E-04	1.02	0.243
DPAGT1	0.25889	0.00055	1.27	5E-06	1.31	3E-08	2.55	2E-07	1.15	0.003
FABP5	2.09174	9.1E-12	2.88	1E-08	1.69	9E-04	7.2	2E-10	1.53	5E-08
FABP6	0.38741	0.19719	1.11	0.838	-1.04	0.309	NA	NA	1.09	0.032
GDE1	-0.1874	0.00614	-1.09	0.082	-1.09	0.019	1.04	0.586	-1.01	0.351
GGPS1	0.14707	0.02283	1.26	1E-09	1.1	0.026	-1.01	0.487	1.07	0.1
GPX5	-0.16	0.64109	-1.11	0.159	NA	NA	NA	NA	1.03	0.201
HACL1	0.1939	0.01393	1.23	2E-06	1.11	0.016	1.3	0.064	1.02	0.302
HMGCR	-0.1994	0.10355	-1.09	0.193	NA	NA	NA	NA	1.03	0.372
HMGCS1	-0.6796	8.2E-07	-1.38	0.007	-1.54	3E-06	-2.92	3E-04	-1.31	0.003
HNF4A	0.11446	0.65965	1.59	0.002	NA	NA	NA	NA	1.1	0.0007
HSD17B1(	0.40213	8.1E-08	1.16	0.0005	NA	NA	NA	NA	-1.03	0.2
INPP5E	0.13011	0.10478	-1.17	0.002	-1.98	1E-13	-1.74	0.006	-1.01	0.287
INS	0.81902	0.44607	1.16	0.034	NA	NA	NA	NA	1.06	0.09
LCAT	-0.4329	0.00031	-1.45	8E-07	NA	NA	-2.37	1E-06	-1.2	7E-08
LDLR	-0.3953	0.01638	-1.45	0.001	-1.22	0.011	-2.72	0.001	-1	0.476
LIPT1	0.08244	0.36068	1.15	0.018	NA	NA	NA	NA	1.06	0.009
LPA	-1.088	4.4E-07	-1.36	0.003	NA	NA	NA	NA	-1.15	2E-05
LRP1	-0.0458	0.76085	-1.08	0.16	1.12	0.09	1.78	0.009	-1.22	0.003
LRP2	-0.8848	0.00022	-1.34	0.137	1.01	0.462	-1.55	0.012	-1.01	0.376
LRP5	-0.0765	0.42904	1.06	0.822	1.23	4E-04	NA	NA	-1.05	0.032
LRP8	0.06425	0.57888	-1.09	0.23	1.11	0.165	-1	0.498	1.1	0.0001
LYPLA1	0.62805	2E-07	1.72	5E-06	1.47	9E-09	3.37	5E-06	1.47	1E-06
LYPLA2	0.40619	9.1E-08	1.28	0.026	1.2	0.009	NA	NA	1.07	0.006
MBTPS1	-0.1997	0.01571	-1.13	0.0008	1.01	0.451	-1.69	0.002	-1.16	0.0009
MBTPS2	-0.0736	0.47056	-1.01	0.446	-1.04	0.202	1.74	0.003	1.04	0.243
MGLL	-0.5752	3.2E-05	-1.4	0.0004	1.08	0.153	-1	0.49	-1.15	0.003
MGST3	-0.5179	1.6E-08	-1.58	2E-08	-1.87	8E-15	-1.57	0.031	-1.29	7E-06
MTTP	-0.9654	1.9E-06	-1.16	0.217	NA	NA	1.03	0.557	1.01	0.411
NR2F2	-0.3142	0.00076	-1.44	9E-05	-1.24	8E-04	1.5	0.02	-1.2	5E-06
OCRL	-0.165	0.09617	-1.03	0.302	1.31	0.002	2.94	1E-08	-1.01	0.447
PAFAH1B:	-0.3708	2.9E-06	-1.37	3E-05	-1.43	6E-10	-1.27	0.024	-1.18	0.004
PCSK9	-1.5693	4.3E-10	-1.3	0.238	-1.04	0.225	-2.04	7E-05	1.05	0.07
PDE3A	-0.4847	0.00919	-1.01	0.478	NA	NA	NA	NA	-1.34	0.003
PEMT	0.13171	0.08795	1.26	0.003	1.64	2E-12	1.35	0.052	-1.01	0.408
PHYH	0.00622	0.93929	-1.01	0.427	1.14	0.027	1.71	0.011	-1.06	0.071
PITPNA	-0.0134	0.8624	-1.06	0.144	-1	0.503	-1.33	0.029	-1.01	0.437
PITPNB	-0.2623	1.7E-05	-1.17	0.005	-1.02	0.369	NA	NA	-1.09	0.04
PITPNM1	-0.4834	1.1E-05	1.07	0.727	NA	NA	-1.24	0.035	-1.04	0.062
PLA2G15	0.26779	0.00101	-1	0.478	1.23	0.001	NA	NA	1.04	0.083
PLCB2	0.12206	0.34428	2.4	0.002	-1.02	0.374	-1.03	0.435	1.01	0.353
PLCB4	0.51189	4E-05	1.52	0.0002	1.44	2E-08	-2.49	2E-05	1.34	6E-05
PLCD3	-1.0438	3.2E-17	-1.86	4E-06	NA	NA	NA	NA	-1	0.471
PLCE1	-0.6922	2.6E-09	-1.44	0.0008	NA	NA	1.11	0.283	-1.58	2E-06
PLCG1	0.17184	0.05591	1.07	0.862	1.2	0.002	1.34	0.038	-1.07	0.034
PLCG2	-0.5447	7.7E-05	-1.15	0.061	NA	NA	-1.27	0.117	1	0.509
PLCH1	-0.843	0.00016	-1.54	0.001	-1.12	0.17	1	0.502	-1.05	0.051
PLCL1	-1.7922	5.5E-17	-2	7E-06	-1.51	3E-07	-1.57	0.018	-1.39	6E-05
PLCZ1	-1.3893	1.9E-09	2.3	0.0003	NA	NA	NA	NA	1.02	0.188

PLIN1	0.77234	7.5E-06	1.23	0.843	NA	NA	1.81	0.002	1.05	0.064
PLTP	-0.5075	0.00144	-1.17	0.083	-1.14	0.08	2.02	0.005	-1.06	0.155
PMVK	0.3044	0.00027	-1.08	0.041	1.02	0.363	-1.11	0.256	-1.03	0.079
PNLIPRP1	0.27928	0.32725	1.34	0.739	NA	NA	1.21	0.6	1.07	0.016
PPAP2B	-0.9868	5.1E-12	-1.53	0.0002	-1.88	2E-10	1.42	0.052	-1.35	3E-05
PPARA	-0.3698	0.001	-1.22	1E-05	-1.38	4E-04	-1.39	0.009	-1.15	3E-05
PPARD	-0.2357	0.0003	-1.16	0.0001	1.35	1E-08	-1.04	0.457	-1.03	0.105
PPARG	-0.79	4.4E-05	-1.81	0.0004	-1.17	0.065	-1.11	0.225	-1.25	0.002
PSAP	-0.1349	0.09346	-1.01	0.473	NA	NA	1.26	0.073	-1.09	0.006
PTGIS	-1.3496	1.4E-11	-2.47	7E-07	-1.94	7E-10	1.04	0.636	1.02	0.18
PTGS1	-2.1137	4.3E-24	-2.8	0.0003	1.14	0.026	-1.21	0.143	-1.53	0.0005
RBP3	-0.2364	0.1699	NA	NA	-1	0.53	-1.88	0.025	1.1	0.005
RDH16	-0.4942	0.03922	1.22	0.052	NA	NA	NA	NA	1.01	0.391
SC5DL	NA	NA	1.03	0.595	1.1	0.094	-1.05	0.38	1.03	0.177
SCAP	0.21481	0.00156	-1.14	0.006	NA	NA	-1.18	0.324	-1.01	0.713
SFTPB	-1.2069	7.2E-05	1.01	0.542	-1.13	0.132	NA	NA	1.02	0.271
SGPL1	0.13728	0.12613	1.07	0.808	1.09	0.141	-1.33	0.036	1.05	0.024
SLC27A3	-0.5729	7E-09	-1.33	3E-06	-1.18	0.008	-1.69	0.003	-1.1	5E-06
SLC27A4	0.79612	9E-20	1.23	0.019	NA	NA	NA	NA	1.16	2E-06
SLC27A5	0.63218	2.6E-11	1.34	5E-07	1.31	2E-06	-3.59	0.073	1.04	0.126
SLC27A6	-0.6208	0.01964	-1.26	0.116	-1.19	9E-04	-1.08	0.38	-1.01	0.437
SREBF1	0.45748	0.00036	1.23	0.002	-1.24	4E-04	1.11	0.605	1.08	0.023
SREBF2	-0.182	0.07631	-1.25	6E-05	1.02	0.36	-2.45	1E-04	-1.12	0.017
STARD3	-0.0993	0.18741	-1.06	0.304	NA	NA	-1.01	0.482	-1.06	0.007
SULT1A1	0.28004	0.02178	1.23	0.0004	1.19	7E-04	NA	NA	-1	0.497
SULT1A3	0.63453	8.6E-05	1.16	0.843	1.13	0.016	NA	NA	-1.07	0.013
THRSP	-1.1672	7.3E-06	-1.36	0.043	NA	NA	NA	NA	1.07	0.076
TPP1	0.00523	0.95303	1.03	0.752	NA	NA	NA	NA	1.01	0.427
TPRA1	0.24258	3.3E-05	1.04	0.711	1.12	0.057	NA	NA	-1.03	0.045
TTPA	-0.3246	0.06715	-1.41	0.037	NA	NA	NA	NA	1.01	0.247
UCP3	0.38613	0.00053	1.07	0.816	NA	NA	NA	NA	1.05	0.103
UGT2B10	-0.5	0.41044	2.75	0.001	NA	NA	NA	NA	1.06	0.036
UGT2B7	-2.1845	2.3E-12	-1.83	0.027	1.11	0.115	NA	NA	1.07	0.016
ZNF202	0.01352	0.8422	-1.2	0.068	1.13	0.036	-1.13	0.22	1.01	0.201

Table S2. Univariate and multivariate analyses of clinic pathological factors for disease free.

Risk factors	Univariate analysis			Multivariate analysis		
	HR	P-value	95% CI	HR	P-value	95% CI
ACSS expression<br>low (n = 175)<br>High (n = 174)	0.563	0.013	0.36-0.89	0.575	0.018	0.36-0.91
Age (years)<br>≤ 60 (n = 148)<br>> 60 (n = 201)	1.56	0.06	0.98-2.47			
Gleason score<br>≤7 (n = 186)<br>> 7 (n = 163)	4.72	0.000	2.79-8.00	2.57	0.001	1.50-4.54
PSA<br>≤4 (n = 325)<br>> 4 (n = 24)	2.90	0.007	1.33-6.33			
M<br>M0 (n = 347)<br>M1 (n = 2)	0.049	0.72	0.00-7.30			
N<br>N0 (n = 282)<br>N1 (n = 67)	1.65	0.05	0.99-2.73			
T<br>T2 (n = 114)<br>T3 or T4 (n = 235)	3.83	0.000	1.97-7.47			
Recurrence<br>NO (n = 296)<br>YES (n = 53)	7.51	0.000	4.82-11.70	5.32	0.000	3.30-8.58

Table S3: IRS for IHC

PR1921b	T	Grade	Gleason	IRS_ACSS3	IRS_PLIN3	M821601	T	Grade	Gleason	IRS_ACSS3	IRS_PLIN3
A1	T2	1	2+2	3	10	A1	T2	1--2	3+3	4	10
A3	T2	1	2+3	2	5	A2	T2	1--2	3+2	4	8
A5	T2	2	3+3	6	6	A3	T2	2	3+2	3	5
A8	0	1	2+2	2	10	A4	T2	2--3	4+4	4	7
A10	T2	1	2+2	3	6	A5	T2	2	3+3	7	7
A12	T2	1	2+3	5	8	A6	T2	2	3+4	4	8
A14	T3	1	2+3	6	7	A7	T2	2--3	4+4	6	8
A16	T3	2	3+3	4	5	A8	T3	2	4+3	4	11
B1	T2	3	3+4	5	8	A9	T3	1--2	3+2	5	9
B3	T3	3	4+4	2	9	A10	T2	3	4+4	6	10
B6	T2	3	3+5	8	5	B1	T2	2	4+3	4	10
B7	T3	3	5+4	6	7	B2	T2	2	4+3	4	12
B10	T2	3	4+4	6	5	B3	T2	1	2+3	6	8
B12	T4	2	3+3	7	7	B4	T3	2	3+3	8	4
B13	T3	3	4+5	4	7	B5	T3	2	3+3	7	5
B16	T2	3	3+4	5	8	B6	T2	2	3+3	4	8
C1	T2	3	3+4	3	4	B7	T2	2--3	4+5	6	9
C3	T2	3	3+4	6	6	B8	T2	1--2	3+2	9	5
C5	T2	3	3+5	6	4	B9	T2	2--3	4+4	4	8
C7	T2	3	3+4	4	9	B10	T2	2--3	4+4	3	4
C9	T2	0	0	3	7	C1	T3	2--3	4+4	6	7
C11	T2	0	0	4	7	C2	T2	3	5+5	7	7
C13	T2	2	3+4	8	5	C3	T2	3	5+5	4	8
C16	T3	2	3+4	5	11	C4	T3	2	4+4	5	8
D1	T3	2	3+4	8	5	C5	T2	2	3+4	3	8
D3	0	3	3+5	4	8	C6	T2	3	5+4	6	6
D5	0	2	3+4	3	9	C7	T3	3	4+5	4	6
D7	T1	2	3+3	4	8	C8	T3	3	4+5	5	6
D9	T2	3	4+4	2	9	C9	T3	2--3	4+4	5	8
D11	T3	3	3+4	3	9	C10	T2	2--3	4+4	5	10
D13	T3	3	4+5	6	5	D1	T3	1--2	3+2	6	8
D15	T2	3	4+5	4	6	D2	T3	2--3	4+4	4	12
E1	T2	2	4+3	7	6	D3	T2	2--3	5+4	4	7
E3	T2	2	4+3	6	7	D4	T3	2--3	5+4	6	9
E5	T2	2	4+3	7	6	D5	T2	2--3	4+4	8	10
E7	T2	2	4+3	4	9	D6	T2	2--3	4+5	5	8
E10	T4	3	4+5	4	6	D7	T2	2--3	4+4	5	9
E12	T2	2	4+3	4	3	D8	T3	2--3	4+5	7	10
E13	T2	2	4+3	5	8	D10	T2	2--3	4+5	4	10
E16	T2	2	4+4	4	11	E1	T4	3	5+4	5	6
F1	T3	3	4+4	6	6	E2	T2	3	5+5	4	9
F3	T3	3	4+4	3	4	E3	T3	2--3	4+5	5	8
F5	T3	3	4+4	7	6	E4	T2	2--3	5+4	6	5
F7	0	3	4+4	3	9	E5	T3	3	5+5	6	8
F10	0	3	4+4	3	10	E6	T2	3	5+4	7	6
F12	0	2	4+3	4	9	E7	T3	3	5+5	4	8
F14	0	3	4+4	3	11	E8	T3	2--3	4+5	5	9
F15	0	3	4+4	3	10	E9	T3	2--3	5+4	4	4
G1	0	3	4+4	2	8	E10	T3	3	5+5	4	5
G3	0	2	4+3	2	9	F1	T2	3	5+4	8	5
G4	0	2	4+3	3	8	F2	T3	2--3	5+4	7	7
G6	T3	3	4+4	4	10	F4	T3	2	4+4	9	4
G9	0	0	0	4	12	F5	T4	2--3	4+5	8	5
G11	T2	2	4+3	8	8	F6	T3	3	5+5	4	9
G13	T2	3	5+5	5	6	F7	T3	2	4+4	6	6
G15	T3	3	5+5	3	10	F8	T2	1	3+2	7	5

H2	T3	3	5+5	5	8	F9	T3	2	4+4	5	4
H4	T2	3	5+5	7	7	PRC1021					
H6	T2	3	5+5	3	6	A6	T2	II	7	8	5
H8	T2	3	5+5	4	9	A7	T1	II	6	8	4
H10	T2	3	5+5	6	12	A8	T2	III	9	6	10
H12	T2	3	5+5	9	3	A9	T2	II	4	8	8
H14	T3	3	5+5	3	12	A10	T2	I	4	6	8
H15	T2	3	5+5	4	12	A11	T2	III	6	6	9
I2	T3	3	5+5	3	9	A12	T2	I	4	4	7
I4	T3	3	5+4	5	8	A13	T2	II	7	5	9
I6	T2	3	5+5	1	8	B1	T2	III	9	3	6
I7	T4	3	5+4	5	6	B2	T2	III	8	7	9
I9	T3	3	5+5	7	8	B3	T3	III	10	5	9
I11	0	3	5+5	3	9	B4	T2	II	6	5	8
I13	0	3	5+5	4	9	B5	T2	III	9	4	7
I15	0	3	5+5	3	11	B6	T2	III	9	5	6
J1	0	3	5+5	5	10	B7	T2	II~III	8	7	5
J3	0	3	5+4	3	9	B8	T1	II~III	8	3	5
J5	0	3	5+4	4	11	B9	T2	III	8	7	4
J7	0	3	4+5	3	10	B10	T2	I	4	5	8
J9	0	3	5+5	4	10	B11	T2	III	8	2	8
J11	0	3	5+5	4	11	B12	T2	II	6	3	9
J13	0	3	5+5	4	10	B13	T2	II	5	4	8
J15	0	3	5+5	5	10	C1	T2	I~II	4	5	8
ProA150CS01						C2	T2	III	8	6	9
A01	T2	2	3+4=7	4	8	C3	T1	III	8	4	8
A03	T2	2	3+4=7	4	8	C4	T2	III	7	3	8
A05	T2	2	3+4=7	4	8	C5	T2	I	4	6	8
A07	T2	2	3+4=7	3	7	C6	T2	III	9	7	8
A09	T2	2	3+4=7	6	8	C7	T2	III	8	4	9
A11	T2	2	3+4=7	5	9	C9	T3	II	7	5	8
A13	T2	2	3+4=7	2	7	C10	T2	III	8	4	8
A15	T2	3	4+3=7	3	9	C11	T1	III	8	3	9
B01	T2	3	4+3=7	7	10	C12	T1	II	5	2	10
B03	T2	1	3+3=6	5	8	C13	T1	II	7	4	12
B05	T2	3	4+3=7	9	8	D1	T2	III	8	3	3
B07	T4	1	3+3=6	6	10	D2	T2	II	6	4	8
B09	T4	1	3+3=6	8	10	D3	T2	I	4	7	8
B11	T3	2	3+4=7	4	10	D4	T2	II	7	5	7
B13	T3	2	3+4=7	3	9	D5	T2	III	8	4	8
B15	T3	2	3+4=7	4	10	D6	T2	III	9	6	10
C01	T4	2	3+4=7	7	9	D7	T2	III	9	4	10
C03	T3	2	3+4=7	8	5	D8	T2	III	8	4	8
C05	T3	2	3+4=7	6	7	D9	T2	II	6	3	11
C07	T3	2	3+4=7	5	7	D10	T4	I	4	4	10
C09	T4	2	3+4=7	4	10	D11	T2	III	9	6	8
C11	T3	2	3+4=7	7	9	D12	T2	III	8	4	11
C13	T3	3	4+3=7	5	8	D13	T2	III	8	5	8
C15	T4	3	4+3=7	8	4	E1	T1	III	8	4	8
D01	T4	3	4+3=7	6	8	E2	T2	III	8	4	8
D03	T3	3	4+3=7	2	5	E3	T2	I	3	5	9
D05	T4	3	4+3=7	8	7	E4	T2	II	7	3	10
D07	T3	3	4+3=7	5	6	E5	T2	III	9	4	10
D09	T3	3	4+3=7	6	6	E6	T2	II	7	6	8
D11	T3	3	4+3=7	8	6	E7	T2	III	8	4	10
D13	T3	4?	3+5=8	8	5	E8	T2	II	7	5	8
D15	T4	4	4+4=8	5	7	E9	T2	III	8	7	9
E01	T3	4	4+4=8	5	7	E10	T2	II	7	3	10

E03	T3	4?	5+3=8	3	8	E11	T2	II	6	5	9
E05	T3	5	4+5=9	6	8	E13	T2	II	5	5	10
E07	—	5	4+5=9	4	7	F1	T1	II	7	7	9
E09	—	5	4+5=9	2	9	F2	T2	III	8	5	9
E11	—	5	4+5=9	9	8	F3	T2	I	3	6	10
E13	T3	5	5+4=9	8	11	F4	T2	III	9	4	9
E15	T3	5	5+4=9	4	10	F5	T2	II	7	3	11
F01	T3	5	5+4=9	6	10	F6	T2	II	6	3	11
F03	T3	5	5+5=10	11	3	F7	T2	I	4	3	12
F05	T4	5	5+5=10	6	8	F8	T1	II	7	5	9
F07	—	1	3+3=6	8	6	F9	T2	I	4	3	12
F09	—	2	3+4=7	8	9	F10	T1	II	6	4	8
F11	T3	2	3+4=7	6	9	F11	T4	II	5	5	11
F13	T3	3	4+3=7	9	5	F12	T2	I	3	5	10
F15	T3	3	4+3=7	5	9	F13	T4	III	8	6	9
G01	T4	4?	3+5=8	3	12	G1	T2	II	7	8	8
G03	T3	5	5+4=9	5	8	G2	T1	II	7	6	7
G05	T2	1	3+3=6	6	9	G3	T2	III	8	5	8
G06	T2	2	3+4=7	9	5	G4	T2	II	5	5	8
G07	T2	2	3+4=7	5	8	G5	T3	III	8	4	11
G08	T3	1	3+3=6	7	9	G7	T2	III	8	4	11
G09	T3	2	3+4=7	6	8	G8	T2	III	8	5	9
G10	T3	2	3+4=7	4	9	G9	T1	III	9	5	9
G11	T3	2	3+4=7	4	9	G10	T4	III	9	5	10
G12	T3	2	3+4=7	6	6	G12	T4	III	9	4	11
G13	T3	2	3+4=7	5	9	H1	T2	III	9	4	7
G14	T3	2	3+4=7	4	10	H2	T2	II	6	4	9
G15	T3	3	4+3=7	4	10	H3	T2	I	3	5	8
G16	T3	3	4+3=7	4	10	H4	T2	III	8	3	12
H01	T3	3	4+3=7	6	10	H5	T4	II	6	6	10
H02	T3	3	4+3=7	3	7	H6	T2	II	5	3	11
H03	T3	4?	3+5=8	8	9	H7	T2	II	7	4	11
H04	T4	4	4+4=8	5	8	H8	T2	III	10	4	10
H05	T4	4	4+4=8	7	10	H9	T2	III	9	4	7
H06	T3	4	4+4=8	6	11	H10	T2	III	7	3	8
H07	T3	4	4+4=8	5	5	H11	T2	III	8	3	12
H08	T3	4?	5+3=8	4	8	H12	T2	I	4	4	10
H09	T4	4?	5+3=8	6	12	0190522R80					
H10	T3	4?	5+3=8	4	9	A1	T3a		9	3	10
H11	T3	4?	5+3=8	5	11	A2	T3a		9	4	10
H12	T3	5	4+5=9	4	7	A3	T2c		6	9	5
H13	T3	5	4+5=9	6	10	A4	T2		6	6	8
H14	T3	5	4+5=9	5	8	A5	T2		9	3	11
H15	T3	5	4+5=9	5	10	A6	T3a		7	5	9
H16	T3	5	4+5=9	4	12	A7	T3a		9	5	11
I01	T3	5	5+4=9	4	6	A8	T3a		7	6	11
I02	T4	5	5+4=9	7	9	A10	T3a		7	3	12
I03	T3	5	5+4=9	6	9	C1	T3		7	5	11
I04	T2	5	5+4=9	8	11	C2	T2c		6	5	9
I05	T4	5	5+4=9	4	8	C3	T2a		9	6	8
I06	T3	5	5+4=9	4	8	C4	T2c		7	6	9
I07	T3	3	4+3=7	5	9	C5	T3a		7	6	7
I08	T4	3	4+3=7	6	8	C6	T3a		8	4	9
I09	—	3	4+3=7	5	9	C7	T3a		7	6	8
I10	T3	3	4+3=7	5	8	C8	T3a		10	3	11
I11	T3	3	4+3=7	7	9	C9	T3a		6	6	6
I12	T4	4?	3+5=8	7	9	C10	T2c		7	6	5
I13	T3	4	4+4=8	7	8	E1	T3a		9	2	10

I14	T4	4	4+4=8	6	9
I15	T3	4	4+4=8	6	9
I16	T3	4	4+4=8	4	9
J01	T3	5	4+5=9	4	11
J02	T3	5	4+5=9	4	9
J03	T3	5	4+5=9	4	11
J04	T3	5	4+5=9	4	8
J05	T3	5	5+4=9	5	9
J06	T3	5	5+4=9	5	9

E2	T2c	7	5	10
E3	T2b	7	5	9
E4	T2a	6	6	9
E5	T3a	7	2	6
E6	T3a	7	4	8
E7	T2c	7	5	8
E8	T2c	7	6	8
E9	T3a	7	6	7
E10	T3a	7	2	12
G1	T3b	9	4	10
G2	T3a	9	3	10
G3	T2c	6	5	9
G4	T3a	7	3	11
G5	T3a	7	4	5
G6	T3b	7	2	12
G7	T2	7	6	9
G8	T2	7	5	9
G9	T2c	9	6	8
G10	T3a	7	3	9

Figure S1

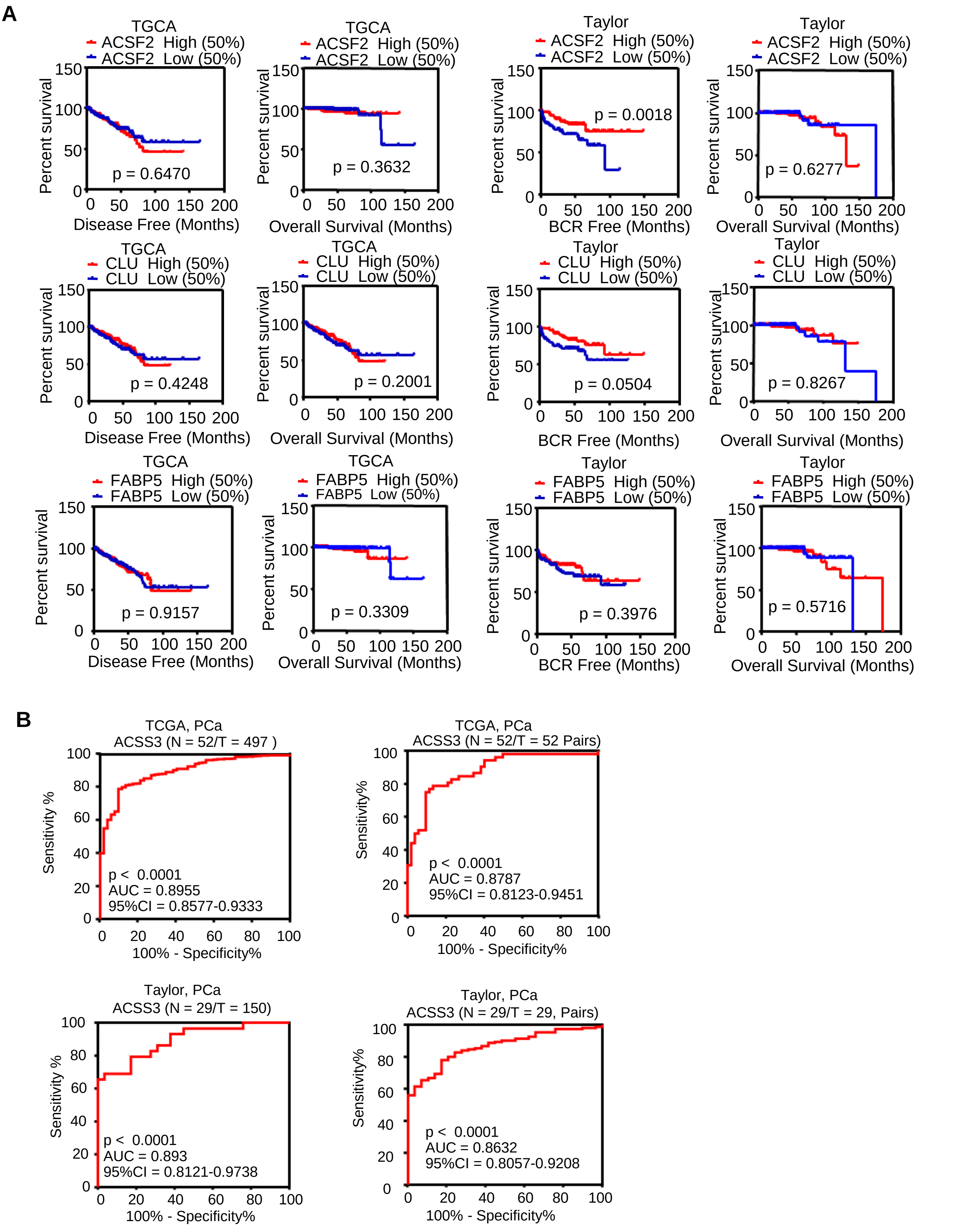
A
TGCA
ACSF2 High (50%)
ACSF2 Low (50%)
Percent survival
Disease Free (Months)
p = 0.6470
TGCA
ACSF2 High (50%)
ACSF2 Low (50%)
Overall Survival (Months)
p = 0.3632
Taylor
ACSF2 High (50%)
ACSF2 Low (50%)
BCR Free (Months)
p = 0.0018
Taylor
ACSF2 High (50%)
ACSF2 Low (50%)
Overall Survival (Months)
p = 0.6277
TGCA
CLU High (50%)
CLU Low (50%)
Disease Free (Months)
p = 0.4248
TGCA
CLU High (50%)
CLU Low (50%)
Overall Survival (Months)
p = 0.2001
Taylor
CLU High (50%)
CLU Low (50%)
BCR Free (Months)
p = 0.0504
Taylor
CLU High (50%)
CLU Low (50%)
Overall Survival (Months)
p = 0.8267
TGCA
FABP5 High (50%)
FABP5 Low (50%)
Disease Free (Months)
p = 0.9157
TGCA
FABP5 High (50%)
FABP5 Low (50%)
Overall Survival (Months)
p = 0.3309
Taylor
FABP5 High (50%)
FABP5 Low (50%)
BCR Free (Months)
p = 0.3976
Taylor
FABP5 High (50%)
FABP5 Low (50%)
Overall Survival (Months)
p = 0.5716
B
TCGA, PCa
ACSS3 (N = 52/T = 497)
Sensitivity %
100% - Specificity%
p < 0.0001
AUC = 0.8955
95%CI = 0.8577-0.9333
TCGA, PCa
ACSS3 (N = 52/T = 52 Pairs)
Sensitivity%
p < 0.0001
AUC = 0.8787
95%CI = 0.8123-0.9451
Taylor, PCa
ACSS3 (N = 29/T = 150)
p < 0.0001
AUC = 0.893
95%CI = 0.8121-0.9738
Taylor, PCa
ACSS3 (N = 29/T = 29, Pairs)
p < 0.0001
AUC = 0.8632
95%CI = 0.8057-0.9208

Figure S2

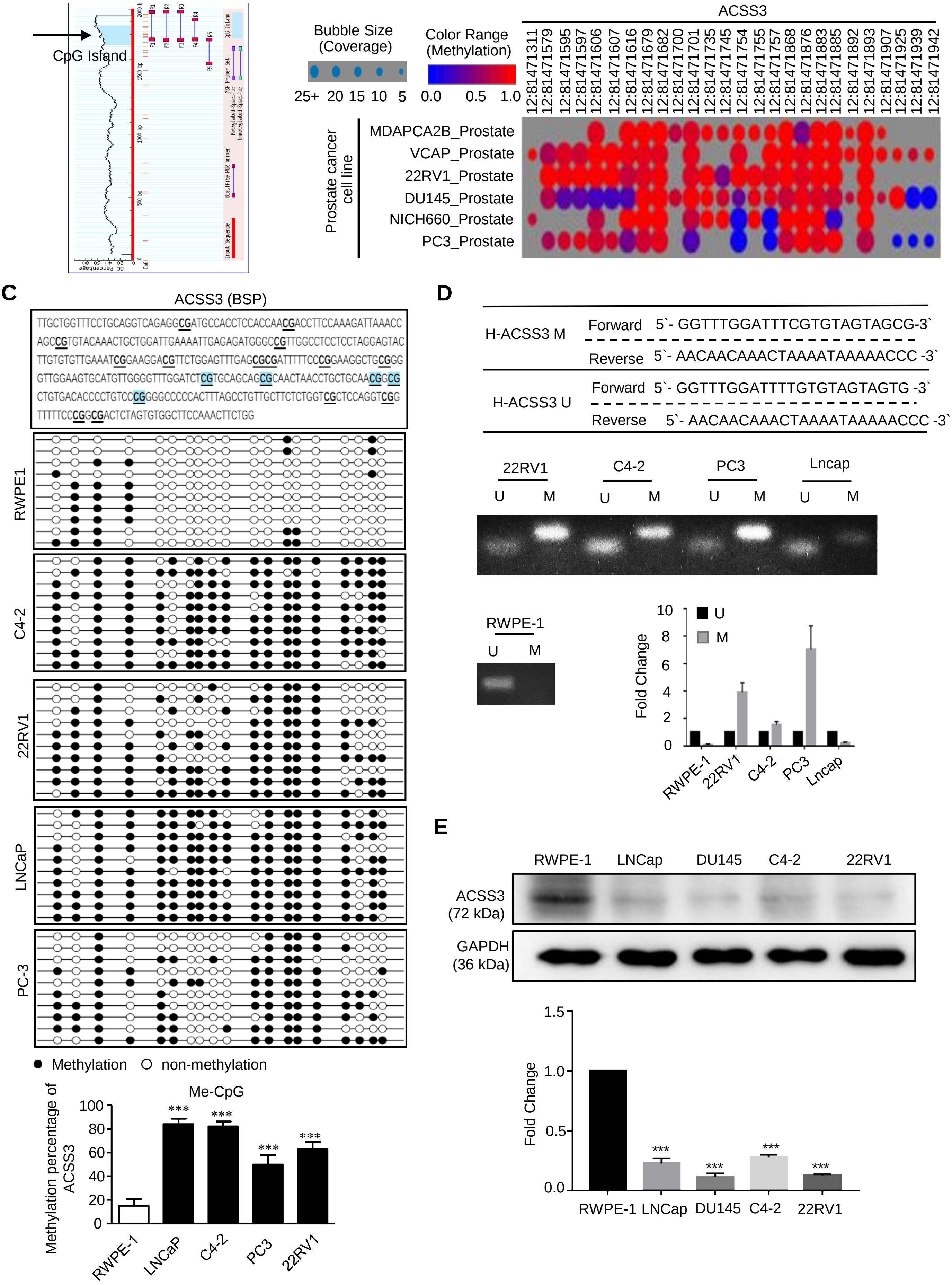

A
CpG Island
B
Bubble Size (Coverage)
25+ 20 15 10 5
Color Range (Methylation)
0.0 0.5 1.0
ACSS3
Prostate cancer cell line
MDAPCA2B_Prostate
VCAP_Prostate
22RV1_Prostate
DU145_Prostate
NICH660_Prostate
PC3_Prostate
C
ACSS3 (BSP)
RWPE1
C4-2
22RV1
LNCaP
PC-3
Methylation
non-methylation
Me-CpG
Methylation percentage of ACSS3
RWPE-1
LNCaP
C4-2
PC3
22RV1
D
H-ACSS3 M
Forward 5`- GGTTTGGATTTCGTGTAGTAGCG-3`
Reverse 5`- AACAACAAACTAAAATAAAAACCC -3`
H-ACSS3 U
Forward 5`- GGTTTGGATTTTGTGTAGTAGTG -3`
Reverse 5`- AACAACAAACTAAAATAAAAACCC -3`
22RV1
C4-2
PC3
Lncap
RWPE-1
U
M
Fold Change
E
RWPE-1
LNCap
DU145
C4-2
22RV1
ACSS3 (72 kDa)
GAPDH (36 kDa)
Fold Change

Figure S2

F

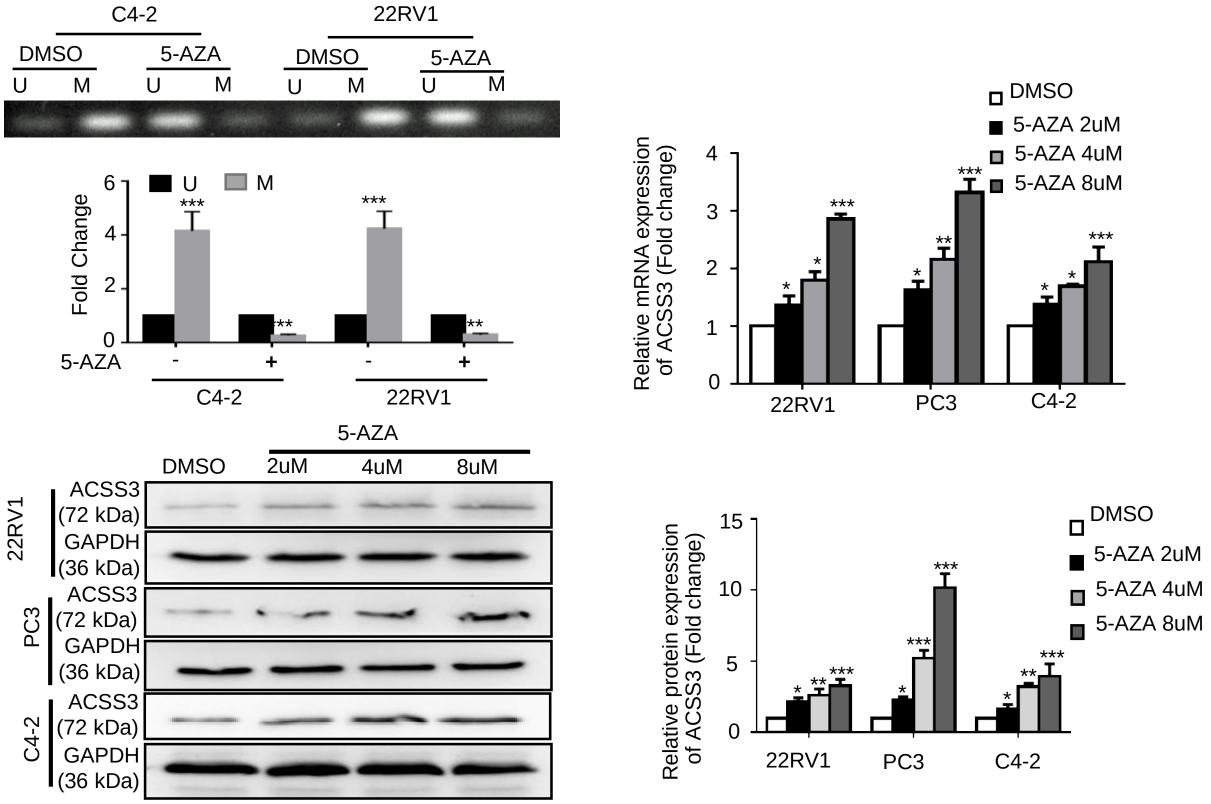
C4-2
22RV1
DMSO
5-AZA
U
M
Fold Change
5-AZA
U
M
***
**
Relative mRNA expression of ACSS3 (Fold change)
DMSO
5-AZA 2uM
5-AZA 4uM
5-AZA 8uM
22RV1
PC3
C4-2
2uM
4uM
8uM
ACSS3 (72 kDa)
GAPDH (36 kDa)
Relative protein expression of ACSS3 (Fold change)

G

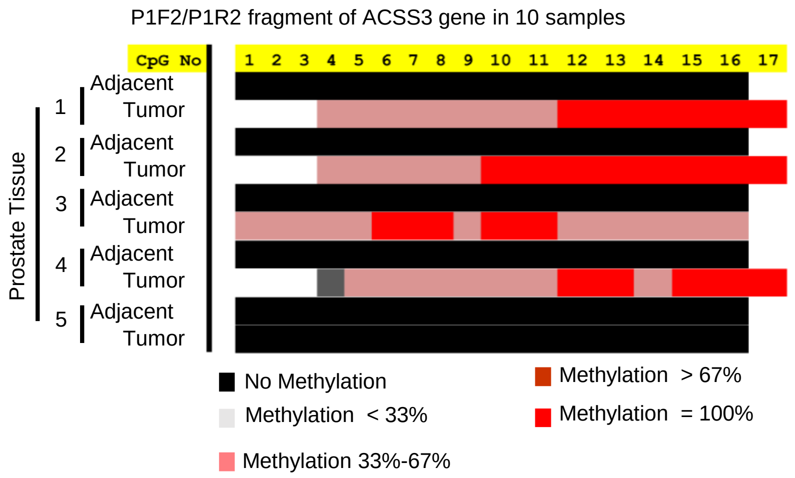
P1F2/P1R2 fragment of ACSS3 gene in 10 samples
CpG No
1 2 3 4 5 6 7 8 9 10 11 12 13 14 15 16 17
Prostate Tissue
1
2
3
4
5
Adjacent
Tumor
No Methylation
Methylation < 33%
Methylation 33%-67%
Methylation > 67%
Methylation = 100%

Figure S3

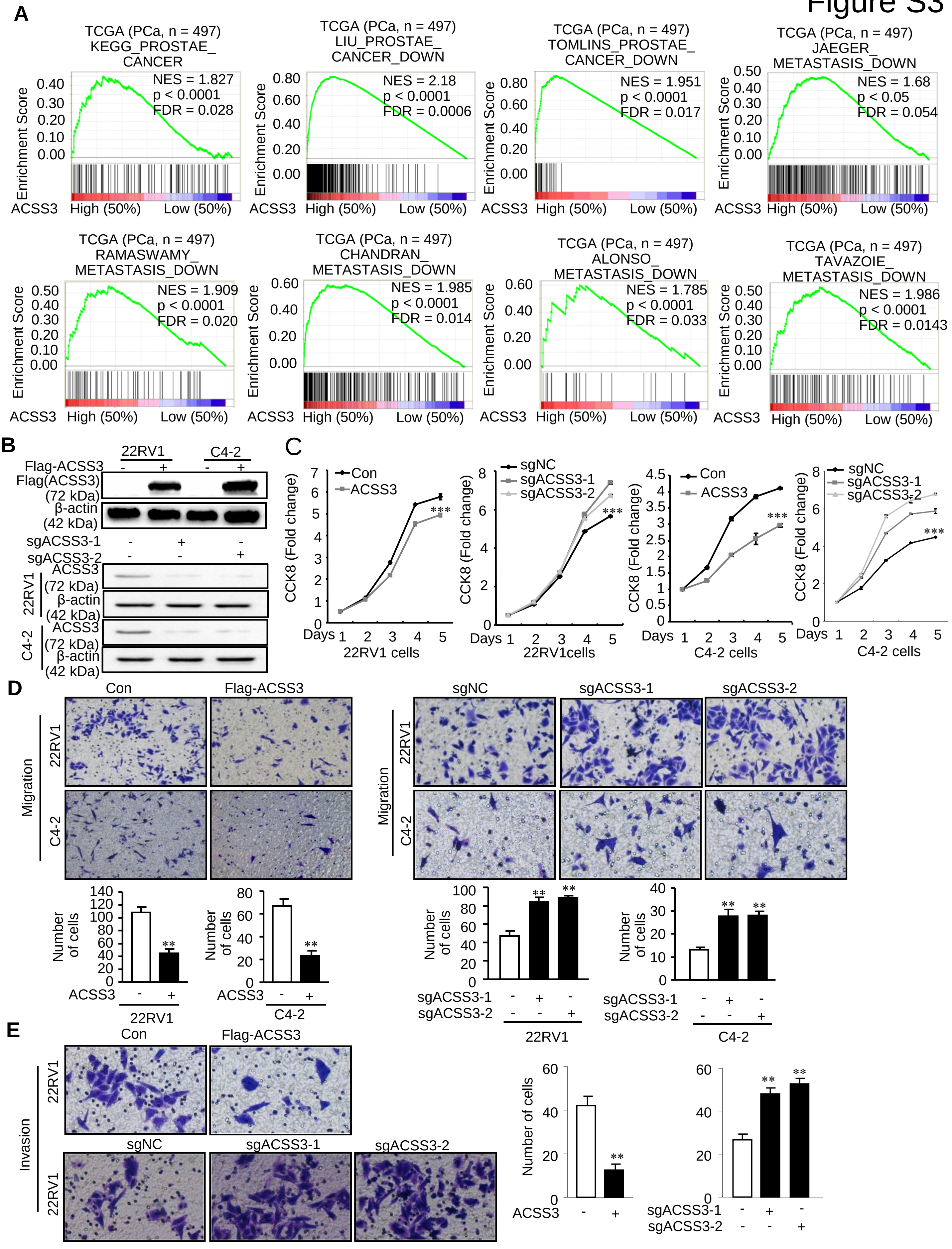
A
TCGA (PCa, n = 497)
KEGG_PROSTAE_CANCER
NES = 1.827
p < 0.0001
FDR = 0.028
LIU_PROSTAE_CANCER_DOWN
NES = 2.18
p < 0.0001
FDR = 0.0006
TOMLINS_PROSTAE_CANCER_DOWN
NES = 1.951
p < 0.0001
FDR = 0.017
JAEGER_METASTASIS_DOWN
NES = 1.68
p < 0.05
FDR = 0.054
RAMASWAMY_METASTASIS_DOWN
NES = 1.909
p < 0.0001
FDR = 0.020
CHANDRAN_METASTASIS_DOWN
NES = 1.985
p < 0.0001
FDR = 0.014
ALONSO_METASTASIS_DOWN
NES = 1.785
p < 0.0001
FDR = 0.033
TAVAZOIE_METASTASIS_DOWN
NES = 1.986
p < 0.0001
FDR = 0.0143
Enrichment Score
ACSS3 High (50%) Low (50%)
B
22RV1
C4-2
Flag-ACSS3
Flag(ACSS3) (72 kDa)
β-actin (42 kDa)
sgACSS3-1
sgACSS3-2
ACSS3 (72 kDa)
C
CCK8 (Fold change)
Con
ACSS3
sgNC
sgACSS3-1
sgACSS3-2
Days
22RV1 cells
22RV1cells
C4-2 cells
***
D
Con
Flag-ACSS3
sgNC
sgACSS3-1
sgACSS3-2
Migration
22RV1
C4-2
Number of cells
**
E
Invasion
Number of cells
ACSS3

Figure S3

F

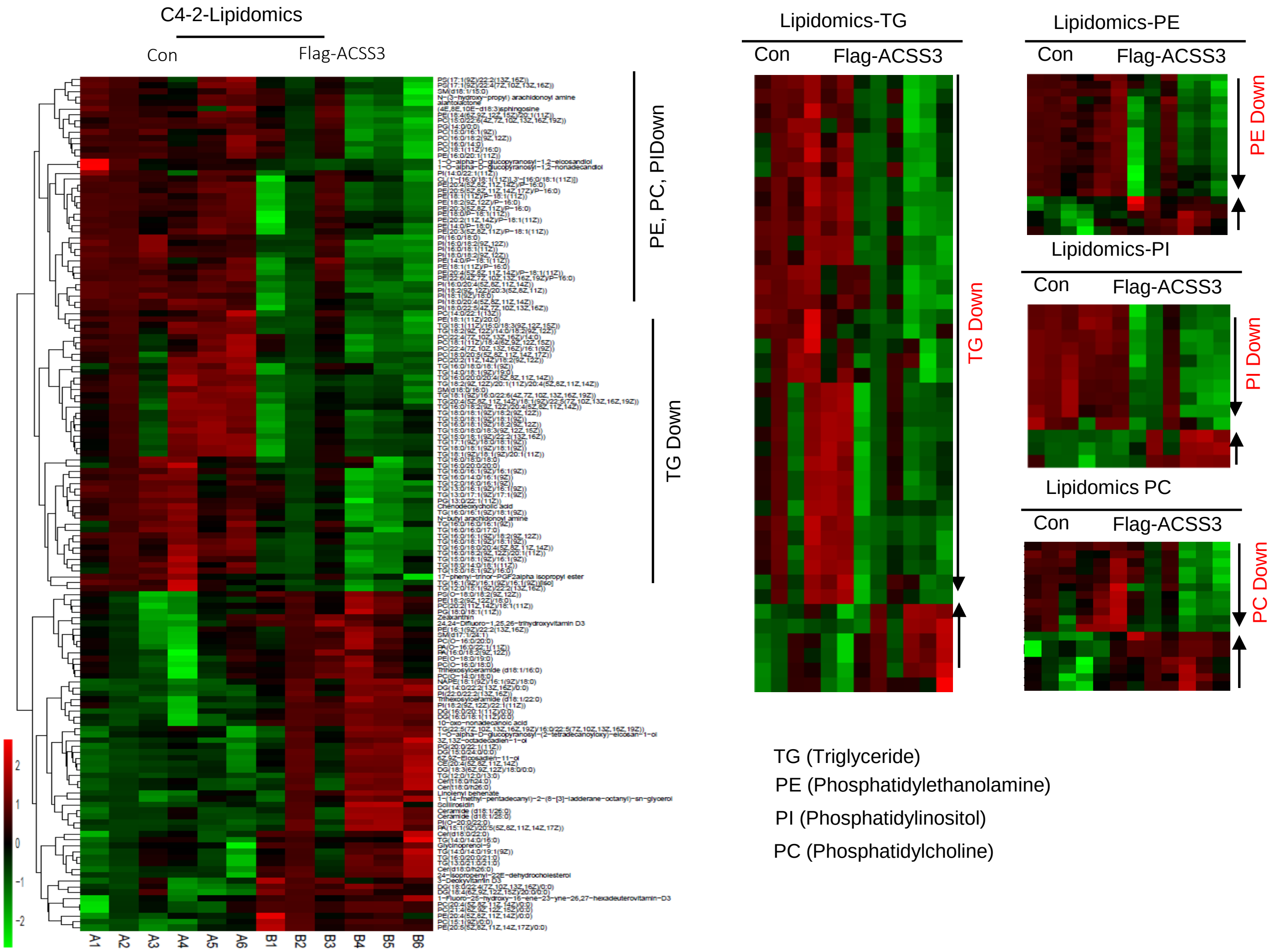
C4-2-Lipidomics
Con
Flag-ACSS3
PE, PC, PIDown
TG Down
A1
A2
A3
A4
A5
A6
B1
B2
B3
B4
B5
B6
2
1
0
-1
-2
Lipidomics-TG
Con
Flag-ACSS3
TG Down
Lipidomics-PE
Con
Flag-ACSS3
PE Down
Lipidomics-PI
Con
Flag-ACSS3
PI Down
Lipidomics PC
Con
Flag-ACSS3
PC Down
TG (Triglyceride)
PE (Phosphatidylethanolamine)
PI (Phosphatidylinositol)
PC (Phosphatidylcholine)

G

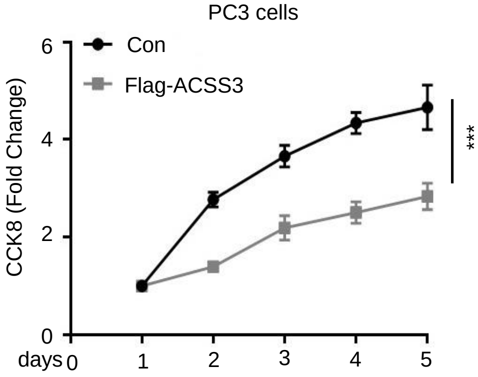
PC3 cells
Con
Flag-ACSS3
CCK8 (Fold Change)
***
6
4
2
0
days 0
1
2
3
4
5

Figure S4

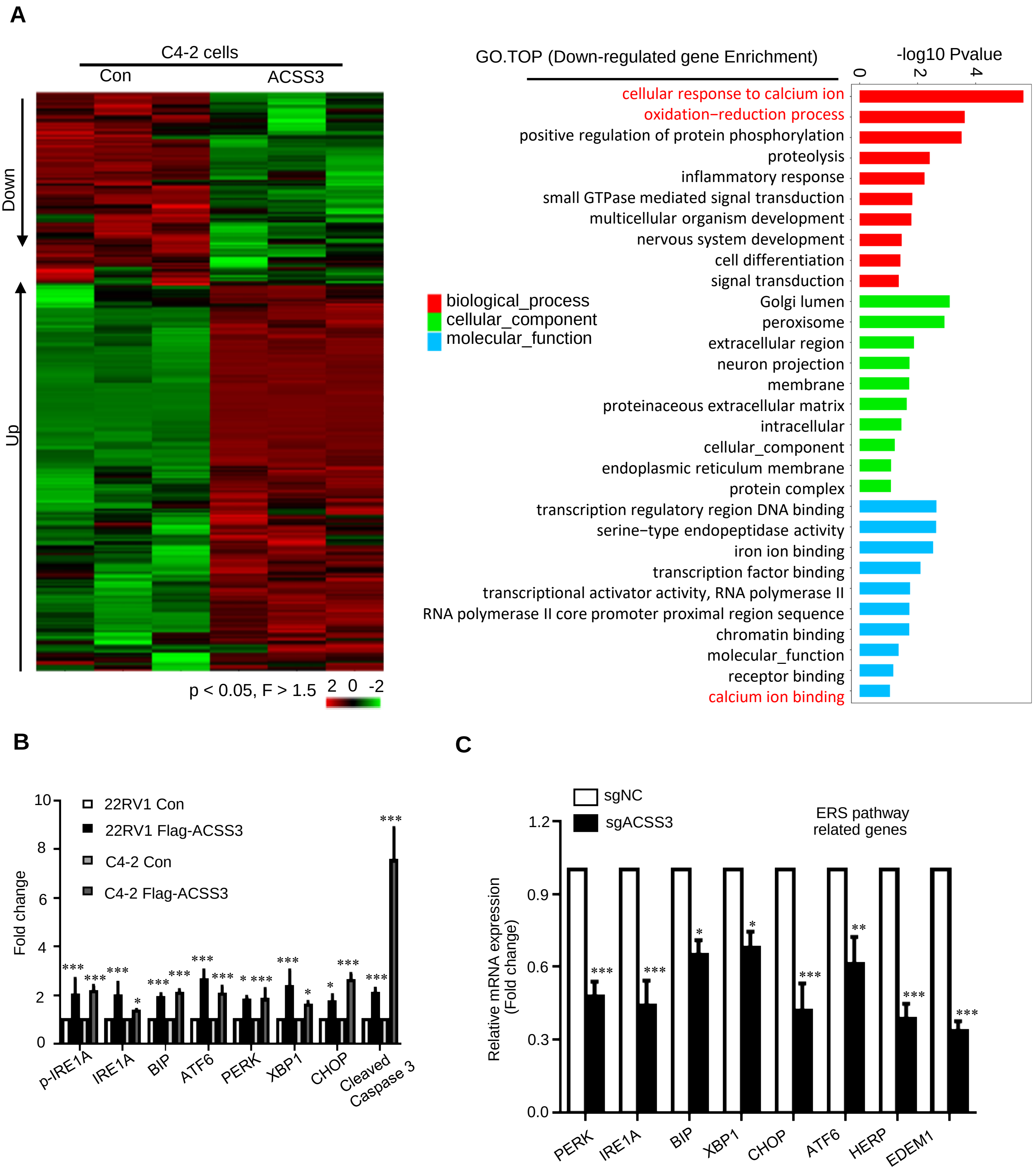

A
C4-2 cells
Con
ACSS3
Down
Up
p < 0.05, F > 1.5
2 0 -2
GO.TOP (Down-regulated gene Enrichment)
-log10 Pvalue
0 2 4
biological_process
cellular_component
molecular_function
cellular response to calcium ion
oxidation−reduction process
positive regulation of protein phosphorylation
proteolysis
inflammatory response
small GTPase mediated signal transduction
multicellular organism development
nervous system development
cell differentiation
signal transduction
Golgi lumen
peroxisome
extracellular region
neuron projection
membrane
proteinaceous extracellular matrix
intracellular
cellular_component
endoplasmic reticulum membrane
protein complex
transcription regulatory region DNA binding
serine−type endopeptidase activity
iron ion binding
transcription factor binding
transcriptional activator activity, RNA polymerase II
RNA polymerase II core promoter proximal region sequence
chromatin binding
molecular_function
receptor binding
calcium ion binding
B
22RV1 Con
22RV1 Flag-ACSS3
C4-2 Con
C4-2 Flag-ACSS3
Fold change
0 2 4 6 8 10
p-IRE1A
IRE1A
BIP
ATF6
PERK
XBP1
CHOP
Cleaved Caspase 3
C
sgNC
sgACSS3
ERS pathway related genes
Relative mRNA expression (Fold change)
0.0 0.3 0.6 0.9 1.2
PERK
IRE1A
BIP
XBP1
CHOP
ATF6
HERP
EDEM1

Figure S4

D

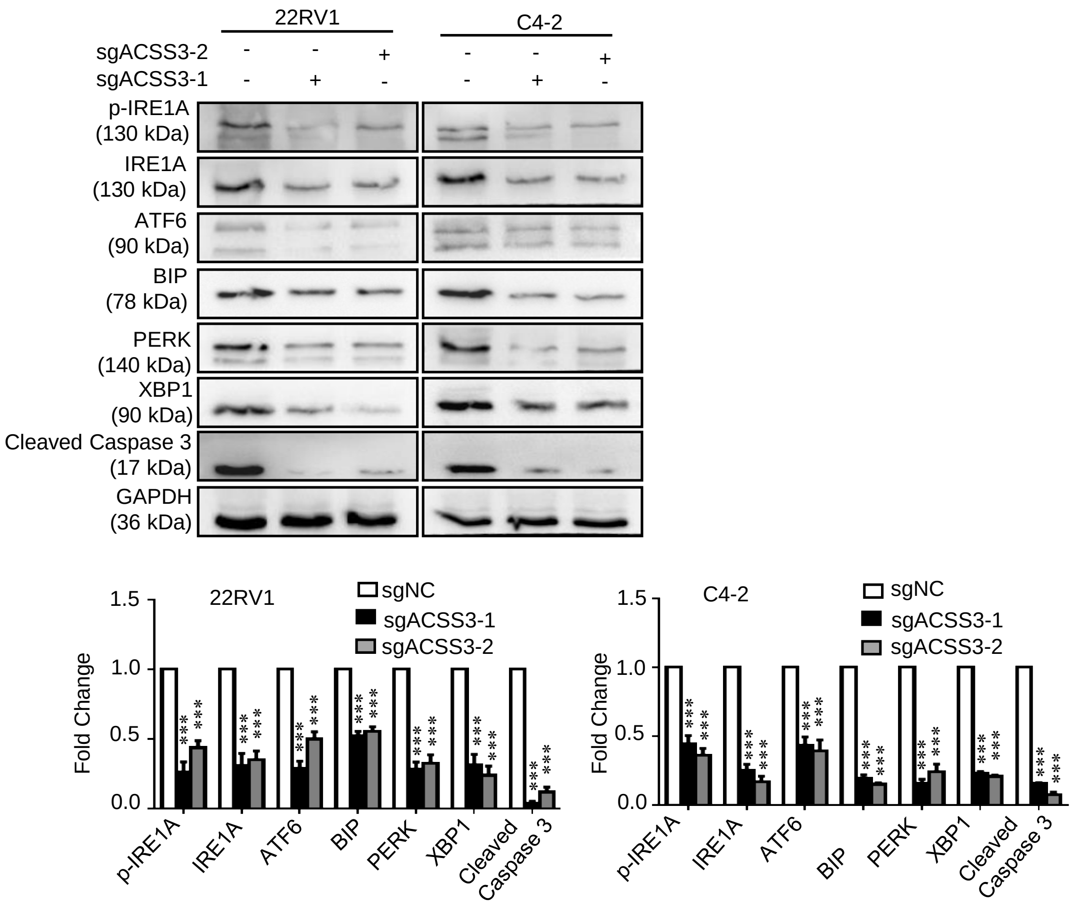
22RV1
C4-2
sgACSS3-2
sgACSS3-1
p-IRE1A (130 kDa)
IRE1A (130 kDa)
ATF6 (90 kDa)
BIP (78 kDa)
PERK (140 kDa)
XBP1 (90 kDa)
Cleaved Caspase 3 (17 kDa)
GAPDH (36 kDa)
sgNC
sgACSS3-1
sgACSS3-2
Fold Change
1.5
1.0
0.5
0.0
p-IRE1A
IRE1A
ATF6
BIP
PERK
XBP1
Cleaved Caspase 3

E

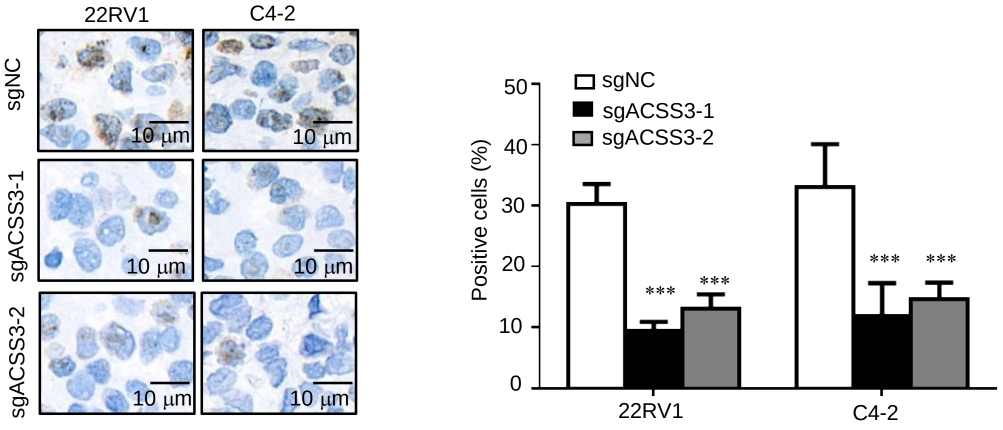
22RV1
C4-2
sgNC
sgACSS3-1
sgACSS3-2
10 μm
Positive cells (%)
50
40
30
20
10
0
***

Figure S5

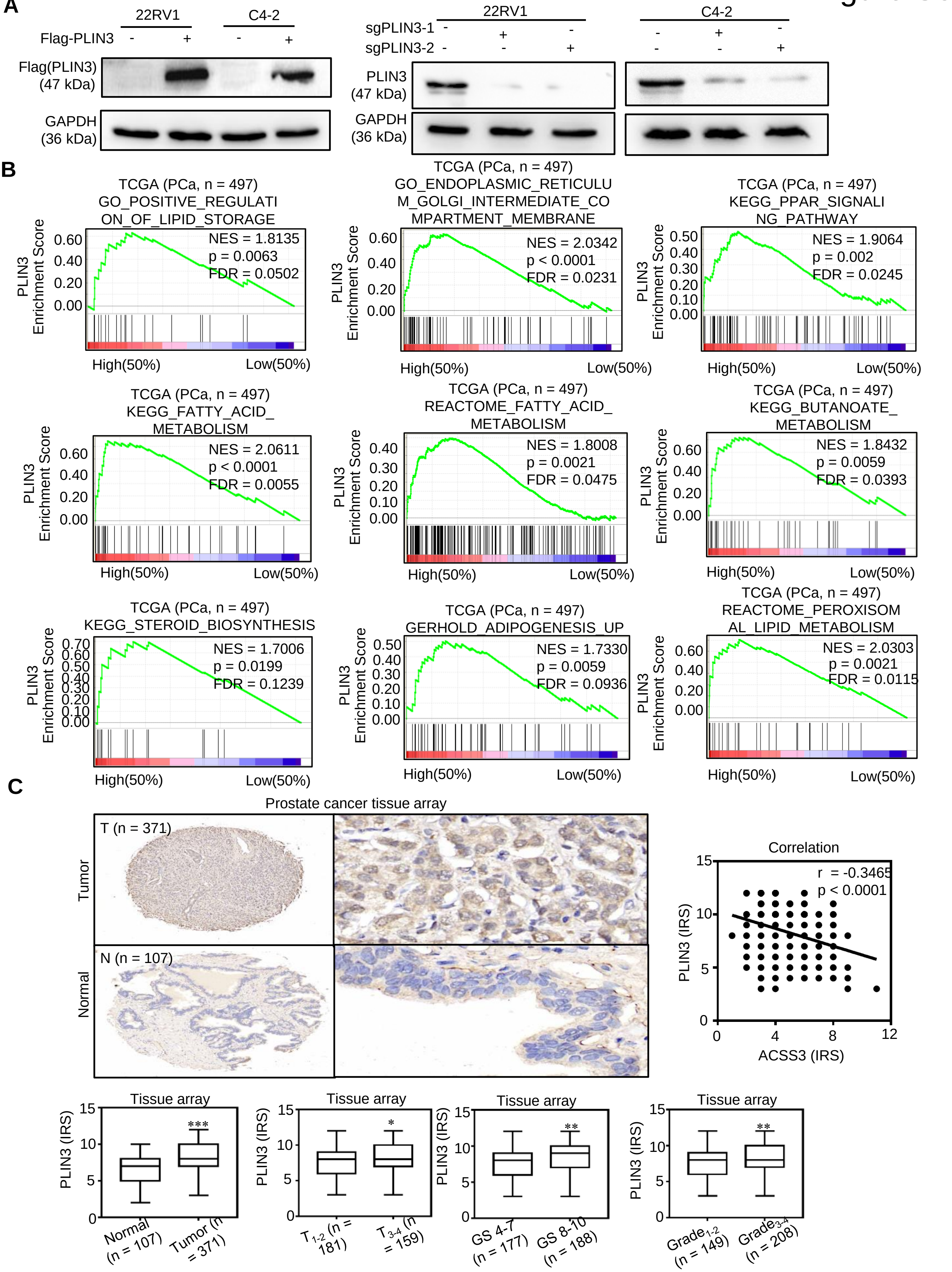

A
22RV1
C4-2
Flag-PLIN3
Flag(PLIN3) (47 kDa)
GAPDH (36 kDa)
sgPLIN3-1
sgPLIN3-2
PLIN3 (47 kDa)
B
TCGA (PCa, n = 497)
GO_POSITIVE_REGULATION_OF_LIPID_STORAGE
NES = 1.8135 p = 0.0063 FDR = 0.0502
GO_ENDOPLASMIC_RETICULUM_GOLGI_INTERMEDIATE_COMPARTMENT_MEMBRANE
NES = 2.0342 p < 0.0001 FDR = 0.0231
KEGG_PPAR_SIGNALING_PATHWAY
NES = 1.9064 p = 0.002 FDR = 0.0245
KEGG_FATTY_ACID_METABOLISM
NES = 2.0611 p < 0.0001 FDR = 0.0055
REACTOME_FATTY_ACID_METABOLISM
NES = 1.8008 p = 0.0021 FDR = 0.0475
KEGG_BUTANOATE_METABOLISM
NES = 1.8432 p = 0.0059 FDR = 0.0393
KEGG_STEROID_BIOSYNTHESIS
NES = 1.7006 p = 0.0199 FDR = 0.1239
GERHOLD_ADIPOGENESIS_UP
NES = 1.7330 p = 0.0059 FDR = 0.0936
REACTOME_PEROXISOMAL_LIPID_METABOLISM
NES = 2.0303 p = 0.0021 FDR = 0.0115
PLIN3 Enrichment Score
High(50%)
Low(50%)
C
Prostate cancer tissue array
T (n = 371)
N (n = 107)
Tumor
Normal
Correlation
r = -0.3465 p < 0.0001
PLIN3 (IRS)
ACSS3 (IRS)
Tissue array
Normal (n = 107)
Tumor (n = 371)
T1-2 (n = 181)
T3-4 (n = 159)
GS 4-7 (n = 177)
GS 8-10 (n = 188)
Grade1-2 (n = 149)
Grade3-4 (n = 208)

Figure S5

D

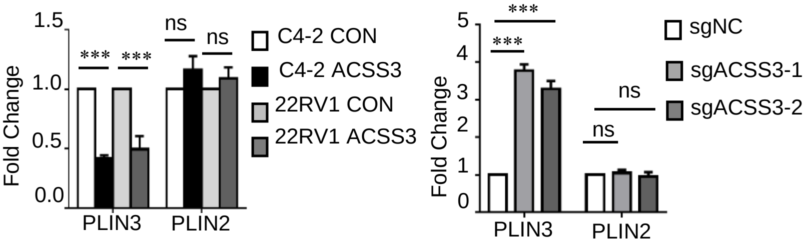

E

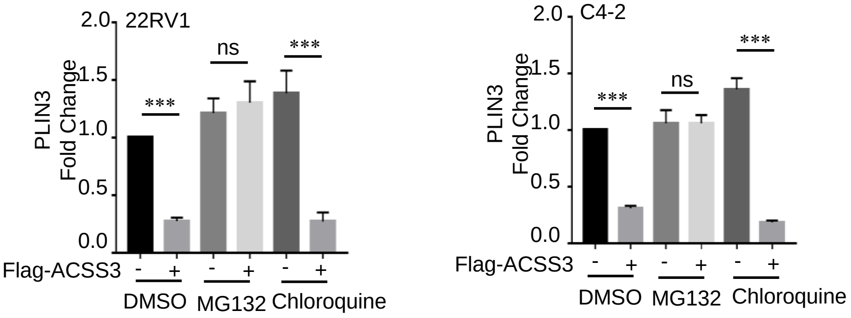

F

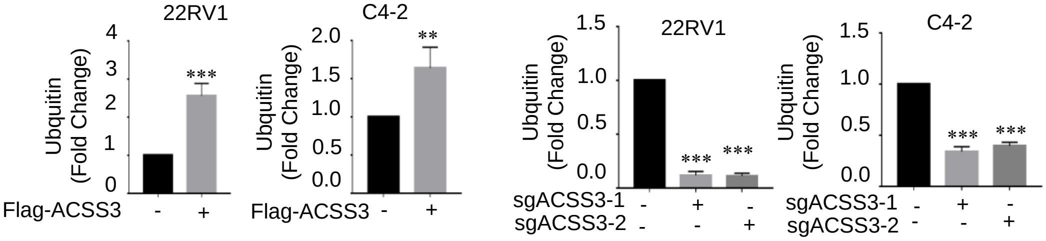

G

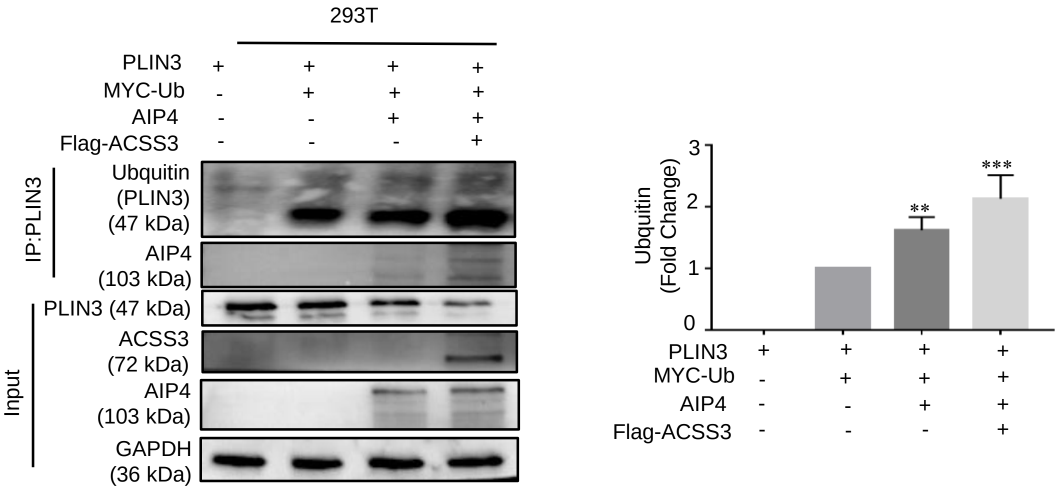

Figure S6

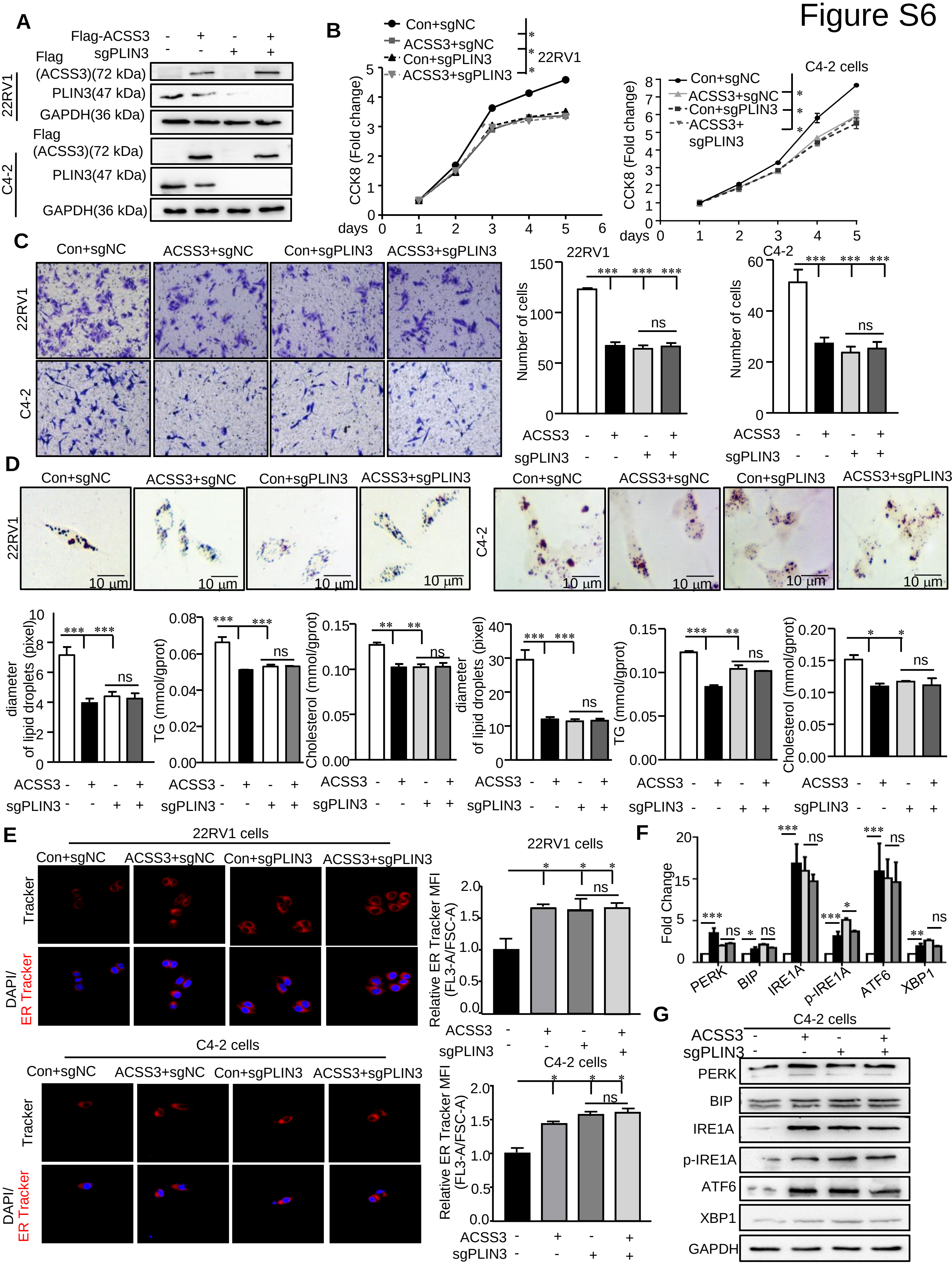
A
Flag-ACSS3
sgPLIN3
22RV1
Flag (ACSS3)(72 kDa)
PLIN3(47 kDa)
GAPDH(36 kDa)
C4-2
B
Con+sgNC
ACSS3+sgNC
Con+sgPLIN3
ACSS3+sgPLIN3
22RV1
C4-2 cells
CCK8 (Fold change)
days
C
Number of cells
ACSS3
sgPLIN3
D
10 μm
diameter of lipid droplets (pixel)
TG (mmol/gprot)
Cholesterol (mmol/gprot)
E
22RV1 cells
C4-2 cells
Tracker
DAPI/ ER Tracker
Relative ER Tracker MFI (FL3-A/FSC-A)
F
Fold Change
PERK
BIP
IRE1A
p-IRE1A
ATF6
XBP1
G
GAPDH

Figure S7

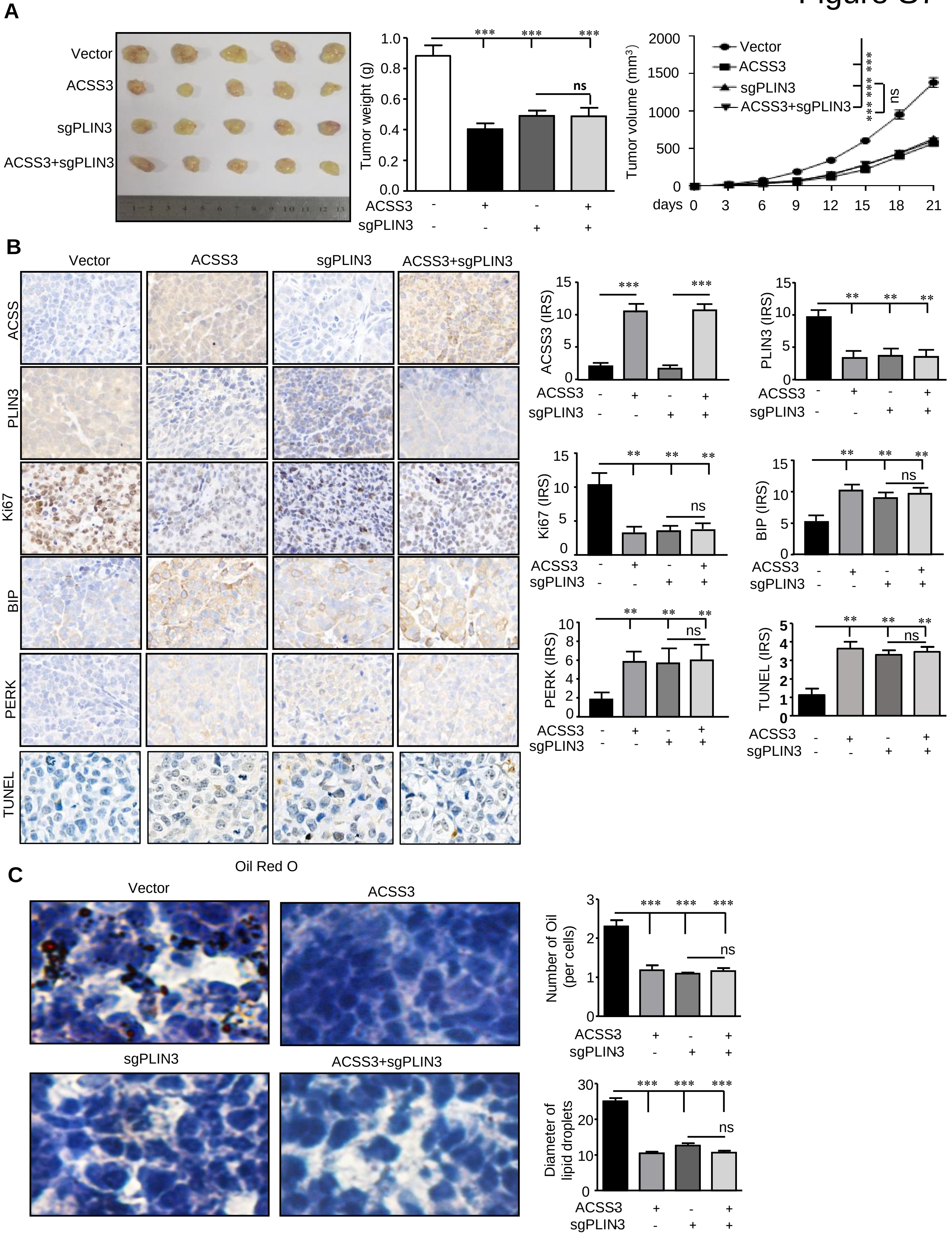
A
Vector
ACSS3
sgPLIN3
ACSS3+sgPLIN3
Tumor weight (g)
ns
ACSS3
sgPLIN3
Tumor volume (mm3)
days
B
ACSS
PLIN3
Ki67
BIP
PERK
TUNEL
ACSS3 (IRS)
PLIN3 (IRS)
Ki67 (IRS)
BIP (IRS)
PERK (IRS)
TUNEL (IRS)
C
Oil Red O
Number of Oil (per cells)
Diameter of lipid droplets

Figure S8

A

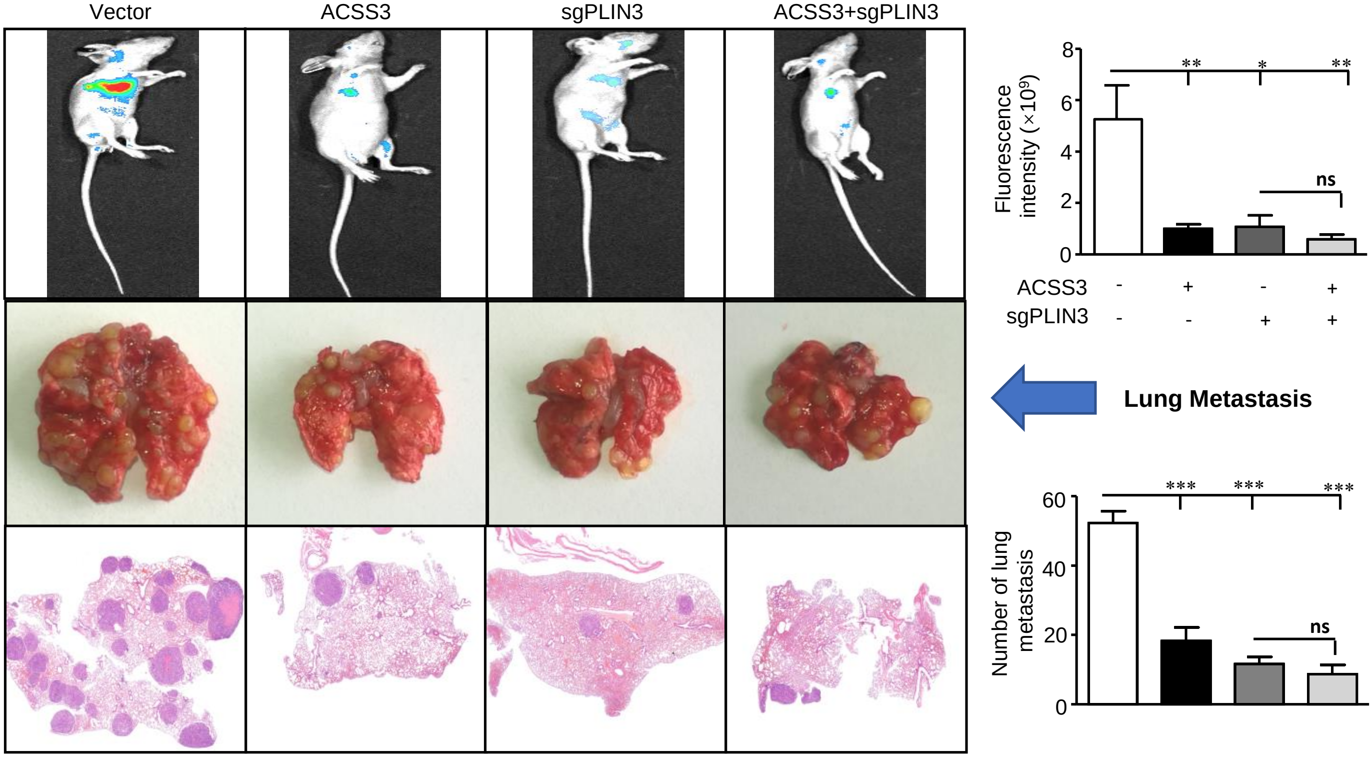

Vector
ACSS3
sgPLIN3
ACSS3+sgPLIN3
Fluorescence intensity (×10⁹)
8
6
4
2
0
**
*
**
ns
ACSS3 - + - +
sgPLIN3 - - + +
Lung Metastasis
Number of lung metastasis
60
40
20
0
***
***
***
ns

B

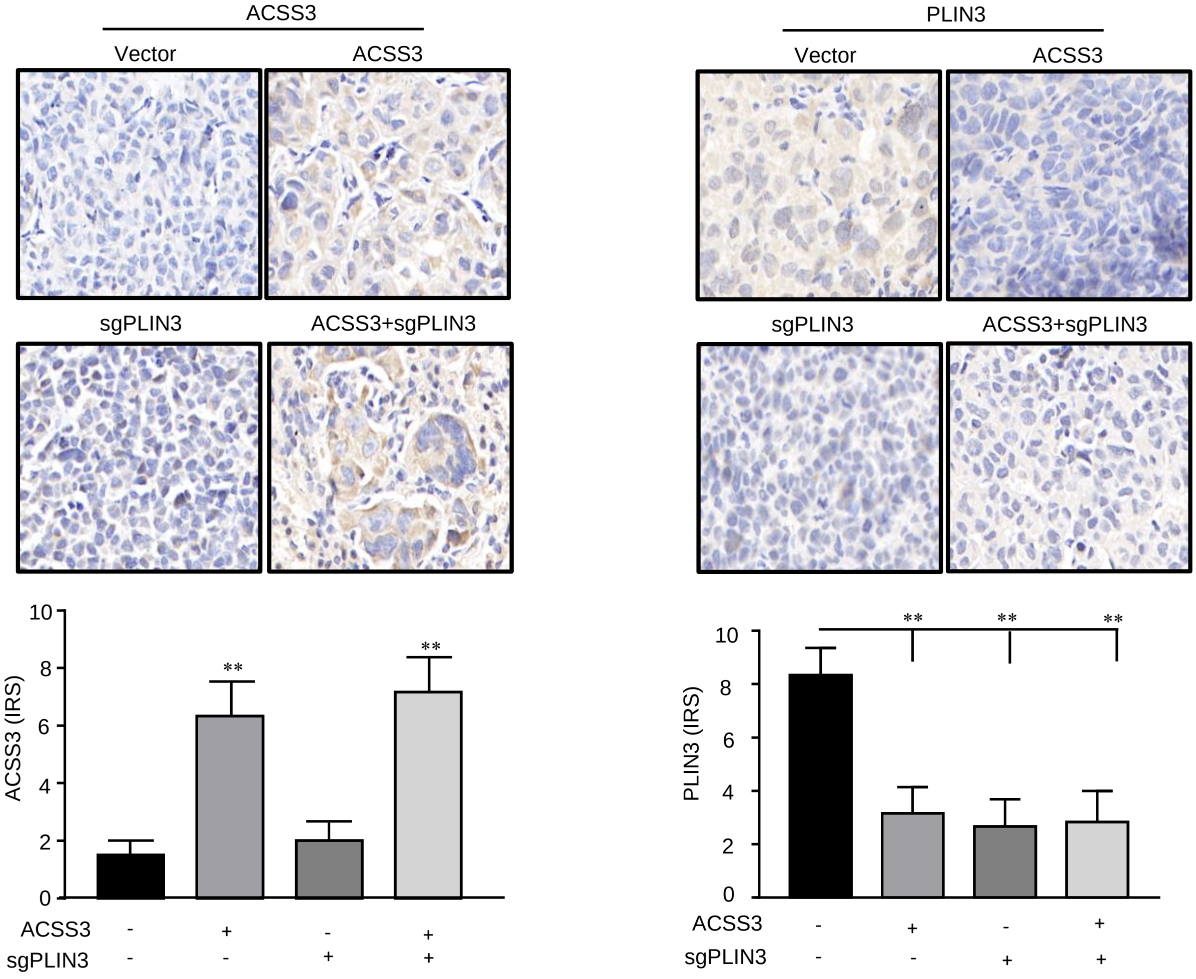

ACSS3
Vector
ACSS3
sgPLIN3
ACSS3+sgPLIN3
PLIN3
Vector
ACSS3
sgPLIN3
ACSS3+sgPLIN3
ACSS3 (IRS)
10
8
6
4
2
0
**
**
ACSS3 - + - +
sgPLIN3 - - + +
PLIN3 (IRS)
10
8
6
4
2
0
**
**
**
ACSS3 - + - +
sgPLIN3 - - + +

Figure S9

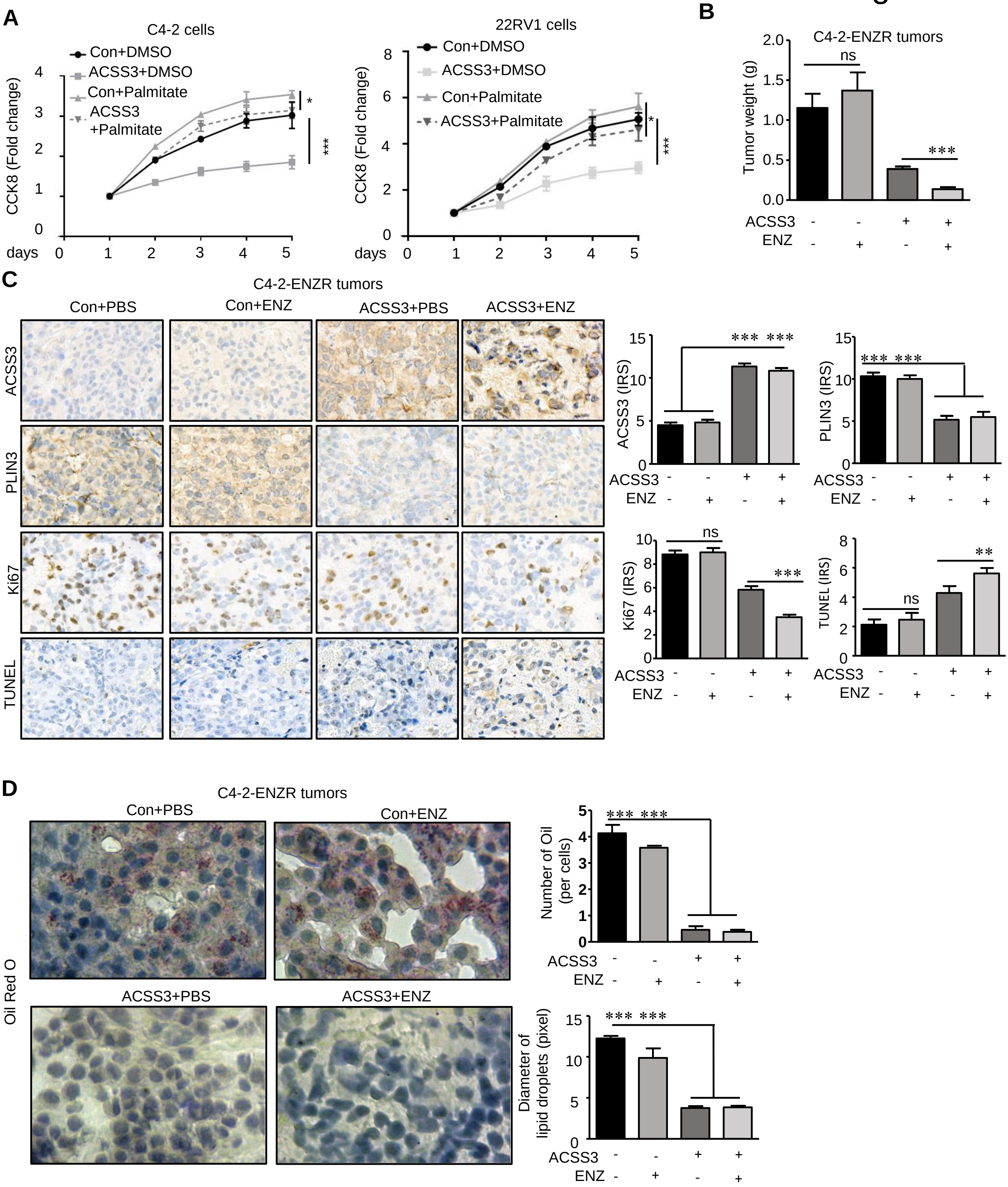

A
C4-2 cells
Con+DMSO
ACSS3+DMSO
Con+Palmitate
ACSS3 +Palmitate
CCK8 (Fold change)
days
22RV1 cells
ACSS3+Palmitate
B
C4-2-ENZR tumors
Tumor weight (g)
ACSS3
ENZ
C
Con+PBS
Con+ENZ
ACSS3+PBS
ACSS3+ENZ
PLIN3
Ki67
TUNEL
ACSS3 (IRS)
PLIN3 (IRS)
Ki67 (IRS)
TUNEL (IRS)
D
Oil Red O
Number of Oil (per cells)
Diameter of lipid droplets (pixel)

Figure S10

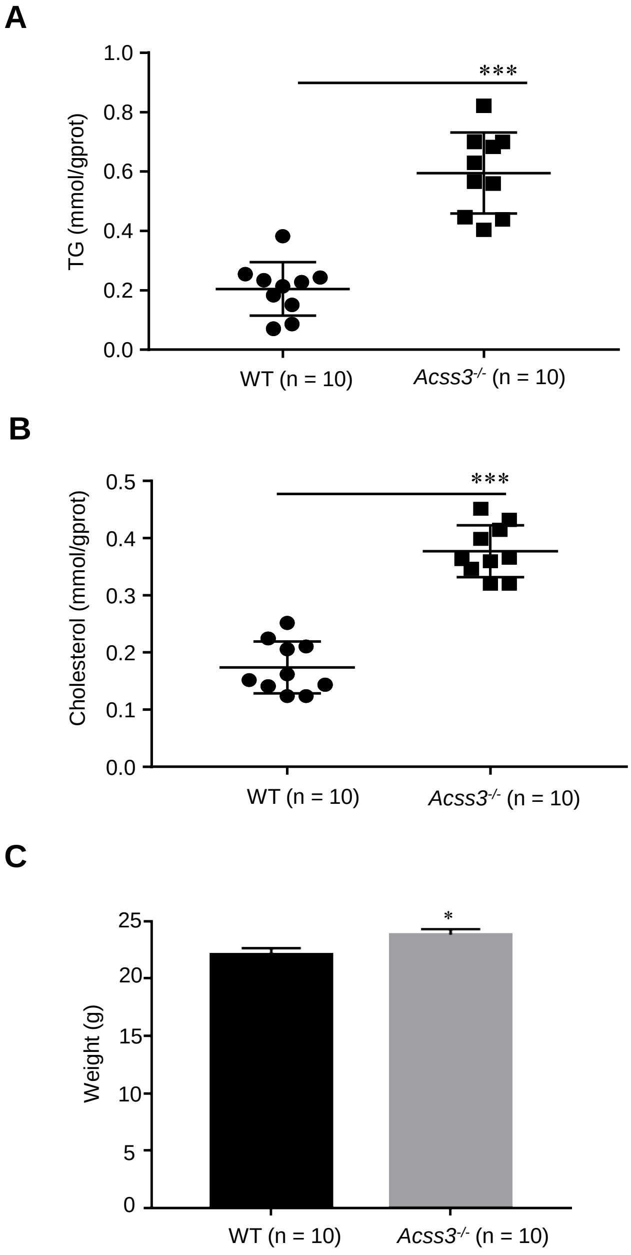
A
TG (mmol/gprot)
1.0
0.8
0.6
0.4
0.2
0.0
***
WT (n = 10)
Acss3-/- (n = 10)
B
Cholesterol (mmol/gprot)
0.5
0.4
0.3
0.2
0.1
0.0
***
WT (n = 10)
Acss3-/- (n = 10)
C
Weight (g)
25
20
15
10
5
0
*
WT (n = 10)
Acss3-/- (n = 10)

Figure S11

A

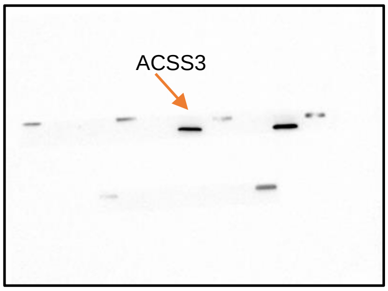

Figure 6E

B

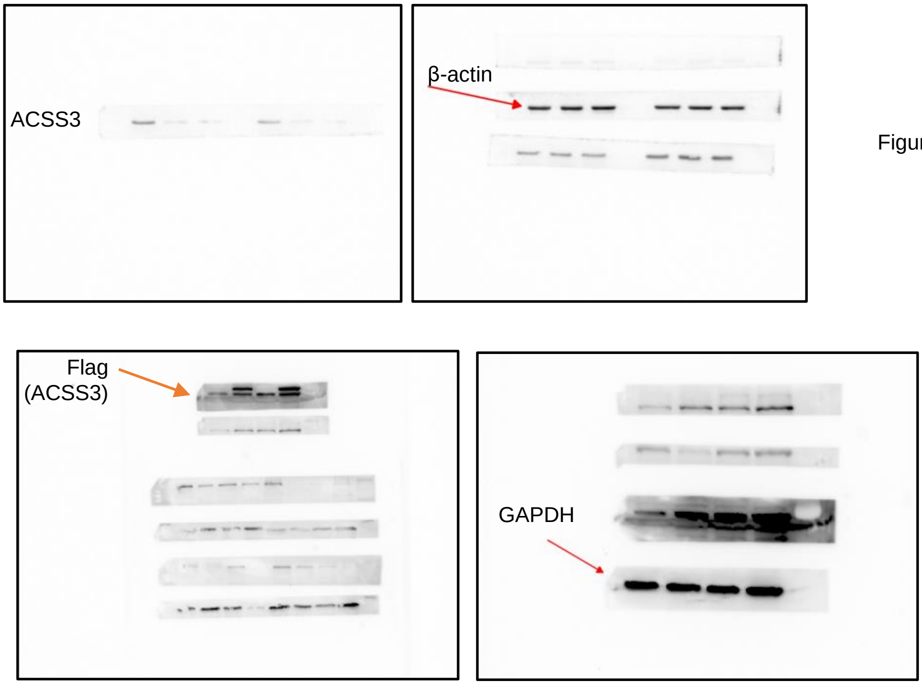

Figure S3B

Figure S3B

C

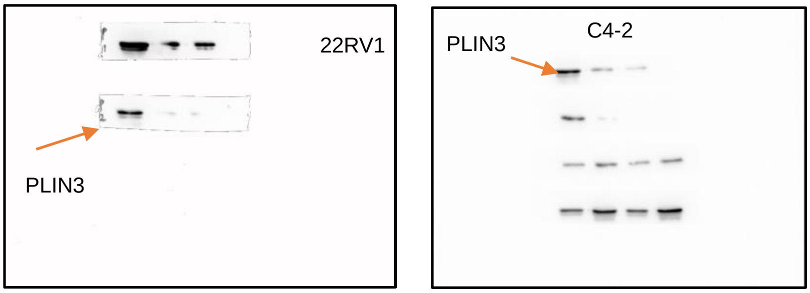

Figure S5A

Supplementary Figure legends

Figure S1. ACSS3 effectively distinguished PCa tissues from normal tissues. (A) Kaplan-Meier analysis in the TCGA and Taylor databases was conducted to determine whether the OS, DFS and BRFS of patients were associated with ACSF2, CLU and FABP5 gene expression in tumors. Gene expression was used to assign patients to the high (above the 50th percentile) or low (below the 50th percentile) expression group. (B) The ROC (receiver operating characteristic) curves of ACSS3 in PCa.

Figure S2. Loss of ACSS3 expression was due to gene promoter methylation. (A) Genomic DNA sequences within the 3-kilobase promoter regions of the ACSS3 gene were analyzed. The results showed that the ACSS3 gene contained CpG-rich regions (CpG islands) within the promoter regions. (B) The publicly available database Cancer Cell Line Encyclopedia (CCLE) was used in bioinformatic analyses to evaluate the methylation status of the ACSS3 promoter in different prostate cancer cell lines. (C) Bisulfite sequencing PCR (BSP) analysis was performed to compare the methylation status of the ACSS3 promoter in prostate cancer cells and in normal prostate epithelial cells. The results represent the mean ± SEM from 3 independent experiments. Student's t test; ***, $p < 0.001$. (D) A methylation-specific PCR (MSP) assay was conducted to verify the methylation sites of the ACSS3 promoter in different prostate cancer cell lines and the normal prostate epithelial cell line. One representative experiment of 3 independent experiments is shown. (E) Western blot was performed to compare the protein levels of the ACSS3 in prostate cancer cells and in normal prostate epithelial cells. The results represent the mean ± SEM from 3 independent experiments. Student's t test; ***, $p < 0.001$. (F)22RV1, PC3 and C4-2 cells were

treated with 5-aza-dC for 5 days. A methylation-specific PCR (MSP) assay was conducted to verify de-methylation after 5-aza-dC. Real-time PCR was profermed to detect the mRNA levels of ACSS3 after 5-aza-dC treatment. ACSS3 protein levels were determined by Western blot analysis in cells. One representative experiment of 3 independent experiments is shown. Student's t test; *, $p < 0.05$; **, $p < 0.01$; ***, $p < 0.001$. (G) BSP analysis was performed to analyze the ACSS3 promoter methylation status in prostate cancer samples.

Figure S3. ACSS3 inhibited tumor cell growth and metastasis. (A) GSEA of ACSS3 mRNA and PCa signaling pathways. $FDR < 25\%$ and $p < 0.05$ were considered significant. (B) ACSS3-overexpressing or ACSS3-knockdown PCa cell lines were generated by transfecting cells with lentiviral overexpression plasmids or sgRNA (CRISPR-Cas9), respectively. ACSS3 protein levels were determined by Western blot analysis. (C) CCK-8 assays were performed to detect PCa cell proliferation. The graphs show the mean ± SEM (n = 6 per group). (D-E) Migration and invasion assays were conducted to evaluate cell migration and invasion. The results represent the mean ± SEM from 3 independent experiments. (F) An LC/MS lipidomic assay was conducted to detect intracellular lipids in C4-2 cells with or without stable overexpression of ACSS3 (n = 6). Only statistically significant changes ($p < 0.05, VIP > 1$) are presented, indicated by the red and green colors in the heat map.(G) CCK-8 assays were performed to detect the ACSS3 proliferation function in PC3 cells. The graphs show the mean ± SEM (n = 6 per group).

Figure S4. ACSS3 promoted ER stress-mediated cell apoptosis in PCa cells. (A) The microarray assays was conducted in C4-2 cells stably overexpressing ACSS3 (n = 3, $p < 0.05$, $F > 1.5$). GO analysis showed that ACSS3 overexpression significantly downregulated the calcium channel-related signaling pathway. (B) Densitometry and statistical analysis of Figure 3D. (C)Real-time PCR analysis was performed to detect the mRNA expression of ER stress

markers. ACSS3 knockout in C4-2 and 22RV1 cells resulted in decreasing mRNA expression of ER stress markers. (D)Western blot analysis was performed to detect the protein expression of ER stress markers. ACSS3 knockout in C4-2 and 22RV1 cells resulted in decreased expression of ER stress markers. One representative experiment of 3 independent experiments is shown. (E) TUNEL assays were performed to detect cell apoptosis. The percentage of apoptotic cells was decreased in ACSS3-knockdown C4-2 and 22RV1 cells. The results represent the mean ± SEM from 3 independent experiments. Student's t test; *, $p < 0.05$; ***, $p < 0.01$; ***, $p < 0.01$.

Figure S5. PLIN3 increased abnormal lipid accumulation and was associated with the clinical features of prostate cancer. And AIP4 was involved in ACSS3 depletion-induced PLIN3 degradation. (A) PLIN3 overexpressing or PLIN3-knockout PCa cell lines were generated by transfecting overexpressing lentivirus or sgRNA (CRISPR-Cas9), respectively. PLIN3 protein levels were determined by Western blot analysis. One representative experiment of 3 independent experiments is shown. (B) GSEA of PLIN3 mRNA in the PCa TCGA dataset. Lipid storage-, fatty acid metabolism-, PPAR- and ER Golgi intermediate compartment membrane-related signaling pathways were highly associated with high PLIN3 expression (above the 50th percentile). *FDR < 25%* and $p < 0.05$ were considered significant. (C) PLIN3 protein expression was analyzed using PCa tissue arrays from 107 normal tissues and 371 tumor tissues. PLIN3 protein levels were significantly higher in PCa tissues than in normal tissues. High PLIN3 expression was negatively correlated with ACSS3 expression and positively correlated with tumor stage and Gleason score. (D) Densitometry analysis of Figure 4B. (E) Densitometry analysis of Figure 4D. (F) Densitometry analysis of Figure 4E and 4F. (G) 293T cells were transfected with PLIN3 with or without Flag-ACSS3, AIP4, and MYC-Ub for 2 days, and total cell lysates were immunoprecipitated with PLIN3 antibody and blotted with the

indicated antibodies. Student’s t test; *, $p < 0.05$; **, $p < 0.01$; ***, $p < 0.001$; ns, no significance.

Figure S6. PLIN3 was required for ACSS3-mediated functions of tumor suppression. (A) C4-2 and 22RV1 cells with stable overexpression of ACSS3 and PLIN3 knockout. ACSS3 and PLIN3 protein levels were determined by Western blot analysis. (B) CCK-8 assays were performed to detect PCa cell proliferation. The graphs show the mean ± SEM (n = 6 per group).(C-E) Migration, oil red staining, measurement the TG and TCHO contents in cells and ER Track were performed to detect PCa cell migration, lipid accumulation, ER stress respectively. The results represent the mean ± SEM from 3 independent experiments. (G-F) Western blot analysis was performed to detect the protein levels of ER stress markers**.** The results represent the mean ± SEM from 3 independent experiments.

Figure S7. ACSS3 inhibited prostate tumor growth *in vivo* by downregulating PLIN3 expression. (A) C4-2 cells with stable overexpression of ACSS3 and/or knockout of PLIN3 were implanted subcutaneously into immunodeficient mice to monitor tumor growth. Tumor size was measured every 3 days. The data are shown as the mean ± SEM for n = 6 separate tumors for each group. Images of tumors dissected from the mice. Tumors were weighed after resection at the end of the experiment. The tumor size (mm3) was plotted against days post tumor cell implantation. One representative experiment of 3 independent experiments is shown. One-way ANOVA followed by Tukey’s multiple comparison test, $\alpha = 0.05$; *, $p < 0.05$; **, $p < 0.01$; ***, $p < 0.001$; ns, no significance. (B) The protein levels of ACSS3, PLIN3, BIP, PERK and Ki67 in tumor tissues were determined by IHC staining. TUNEL assays were performed to detect cell apoptosis. Bar graphs show the mean ± SEM (n = 6 mice per group). (C) Oil red staining was conducted as a visual indicator of intracellular lipids in tumor tissues. Bar graphs show the mean

± SEM (n = 6 mice per group). One-way ANOVA followed by Tukey's multiple comparison test; α = 0.05; ***, $p < 0.001$; ns, no significance.

Figure S8. ACSS3 inhibited prostate tumor metastasis *in vivo* by downregulating PLIN3 expression. (A) C4-2 cells with stable overexpression of ACSS3 and/or knockout of PLIN3 were implanted into the tail veins of immunodeficient mice. In vivo live imaging of the mouse lungs was conducted to determine tumor metastasis. HE staining of nude mouse lungs was conducted to determine tumor metastasis. The graphs show the mean ± SEM (n = 6 mice per group). One representative experiment of 3 independent experiments is shown. One-way ANOVA followed by Tukey's multiple comparison test, α = 0.05; 0.05; *, p < 0.05; **, p < 0.01; ***, p < 0.001; ns, no significance. Bar graphs show the mean ± SEM (n = 6 mice per group). (B) The protein levels of ACSS3 and PLIN3 in tumor tissues were determined by IHC staining. Bar graphs show the mean ± SEM (n = 6 mice per group). One-way ANOVA followed by Tukey's multiple comparison test, α = 0.05; *, $p < 0.05$; **, $p < 0.01$; ***, $p < 0.001$; ns, no significance.

Figure S9. Palmitate could rescue the inhibitory effect of overexpression of ACSS3, and restoration of ACSS3 expression reverted endocrine therapy-resistant CRPC progression *in vivo*. (A) Treatment the cells with palmitate (100 μM), and CCK8 was preformed to test the proliferation of the cells. (B) NCG mice bearing C4-2-ENZR xenografts with stable ACSS3 overexpression were treated with vector control or enzalutamide (10 mg/kg p.o.) for approximately 7 weeks. Tumors were weighed after resection at the end of the experiment. Bar graphs show the mean ± SEM (n = 6 mice per group). One-way ANOVA followed by Tukey's multiple comparison test, α = 0.05; ***, $p < 0.001$; ns, no significance. (B) The protein levels of ACSS3, PLIN3 and Ki67 in tumor tissues were determined by IHC staining. TUNEL assays were performed to detect cell apoptosis. Bar graphs show the mean ± SEM (n = 6 mice per

group). One-way ANOVA followed by Tukey's multiple comparison test, $\alpha = 0.05$; **, $p < 0.01$. (C) Oil red staining was conducted as a visual indicator of intracellular lipids in tumor tissues. Bar graphs show the mean ± SEM (n = 6 mice per group). One-way ANOVA followed by Tukey's multiple comparison test, $\alpha = 0.05$; ***, $p < 0.001$; ns, no significance.

Figure S10. TG and cholesterol contents were increased in the *Acss3*$^{-/-}$ prostate tissues. (A-B) TG contents and cholesterol contents were measured as a quantitative indicator of lipid accumulation in Acss3-/- prostate tissues. (C) The weight of Acss3-/- and control mouse was measured. The graphs show the mean ± SEM (n = 10 mice per group), Student's t test; *, $p < 0.05$; **, $p < 0.01$; ***, $p < 0.001$; NS, no significance.

Figure S11. The original WB band. (A) The original WB band of Figure 6E. (B) The original WB band of Figure S3B. (C) The original WB band of Figure S5A.
